# Divergent degeneration of *creA* antitoxin genes from minimal CRISPRs and the convergent strategy of tRNA-sequestering CreT toxins

**DOI:** 10.1093/nar/gkab821

**Published:** 2021-09-22

**Authors:** Feiyue Cheng, Rui Wang, Haiying Yu, Chao Liu, Jun Yang, Hua Xiang, Ming Li

**Affiliations:** State Key Laboratory of Microbial Resources, Institute of Microbiology, Chinese Academy of Sciences, Beijing, China; College of Life Science, University of Chinese Academy of Sciences, Beijing, China; CAS Key Laboratory of Microbial Physiological and Metabolic Engineering, Institute of Microbiology, Chinese Academy of Sciences, Beijing, China; Non-coding RNA and Drug Discovery Key Laboratory of Sichuan Province, Chengdu Medical College, Chengdu, Sichuan, China; State Key Laboratory of Microbial Resources, Institute of Microbiology, Chinese Academy of Sciences, Beijing, China; CAS Key Laboratory of Microbial Physiological and Metabolic Engineering, Institute of Microbiology, Chinese Academy of Sciences, Beijing, China; CAS Key Laboratory of Microbial Physiological and Metabolic Engineering, Institute of Microbiology, Chinese Academy of Sciences, Beijing, China; Center for Life Science, School of Life Sciences, Yunnan University, Kunming, China; State Key Laboratory of Microbial Resources, Institute of Microbiology, Chinese Academy of Sciences, Beijing, China; College of Life Science, University of Chinese Academy of Sciences, Beijing, China; CAS Key Laboratory of Microbial Physiological and Metabolic Engineering, Institute of Microbiology, Chinese Academy of Sciences, Beijing, China; State Key Laboratory of Microbial Resources, Institute of Microbiology, Chinese Academy of Sciences, Beijing, China; College of Life Science, University of Chinese Academy of Sciences, Beijing, China

## Abstract

Aside from providing adaptive immunity, type I CRISPR-Cas was recently unearthed to employ a noncanonical RNA guide (CreA) to transcriptionally repress an RNA toxin (CreT). Here, we report that, for most archaeal and bacterial CreTA modules, the *creA* gene actually carries two flanking ‘CRISPR repeats’, which are, however, highly divergent and degenerated. By deep sequencing, we show that the two repeats give rise to an 8-nt 5′ handle and a 22-nt 3′ handle, respectively, i.e., the conserved elements of a canonical CRISPR RNA, indicating they both retained critical nucleotides for Cas6 processing during divergent degeneration. We also uncovered a minimal CreT toxin that sequesters the rare transfer RNA for isoleucine, tRNA^Ile^_CAU_, with a six-codon open reading frame containing two consecutive AUA codons. To fully relieve its toxicity, both tRNA^Ile^_CAU_ overexpression and supply of extra agmatine (modifies the wobble base of tRNA^Ile^_CAU_ to decipher AUA codons) are required. By replacing AUA to AGA/AGG codons, we reprogrammed this toxin to sequester rare arginine tRNAs. These data provide essential information on CreTA origin and for future CreTA prediction, and enrich the knowledge of tRNA-sequestering small RNAs that are employed by CRISPR-Cas to get addictive to the host.

## INTRODUCTION

Adaptive immunity in prokaryotes is mediated by CRISPR-Cas systems that defend archaea and bacteria against recurrent invasions of foreign genetic elements, such as viruses and plasmids (1–4). CRISPR is an array of short DNA repeats that are interspaced by non-repetitive DNA segments, known as spacers, derived from invading nucleic acid. CRISPR arrays are typically accompanied by an operon encoding CRISPR-associated (Cas) proteins that functionally interact with CRISPR. CRISPR-Cas systems are highly diversified and to date have been divided into two classes, six types, and 33 subtypes, with class 1 systems being most prevalent across bacterial and archaeal species (5).

A complex of Cas1, Cas2 and, in some cases, also Cas4 mediates the acquisition of new spacers from an invading DNA, a process that is known as CRISPR adaptation (3,6). Transcripts from the CRISPR array contain conserved repeat sequences that are recognized and processed by a Cas nuclease or a host enzyme, thus giving rise to a set of small CRISPR RNAs (crRNAs) (7–10). Mature crRNAs usually carry the conserved repeat-derived sequences (handles) flanking the invader-targeting spacer sequence. Based on the complementarity between the spacer sequence and its cognate sequence (termed protospacer) on the invading DNA/RNA, crRNAs guide a multi-subunit effector complex (class 1) or a single-polypeptide effector (class 2) to inactivate the foreign DNA/RNA, thus protecting the host specifically from the targeted genetic invader (5,11).

To attack a target DNA, the CRISPR effector first recognizes a conserved protospacer adjacent motif (PAM), which is critical for self versus non-self discrimination (12,13). However, in spite of this safeguard, and characteristically of defense systems in general, CRISPR-Cas creates a risk of auto-immunity, when a host DNA fragment is accidentally acquired as a new spacer unit. Indeed, self-targeting spacers have been detected across different types of CRISPR-Cas (14). The activity of CRISPR-Cas also impedes the acquisition of beneficial exogenous genes when targeting their carrier plasmid (or virus), which causes another evolutionary downside of adaptive immunity in prokaryotes (15,16). Therefore, CRISPR-Cas systems impart non-negligible fitness costs on the host, which results in their frequent loss and patchy distribution among prokaryotic species (5). Nevertheless, these systems are represented in ∼40% of bacteria and ∼90% of archaea (5), suggesting that, in addition to the direct benefits as a defense system, CRISPR-Cas could have evolved mechanisms to mitigate its fitness costs on the host.

Our recent study unearthed a diverse set of CRISPR-regulated toxin-antitoxin (CreTA) RNA pairs, which safeguard Cascade complexes, the multi-subunit effectors of type I CRISPR-Cas, by making them addictive to the host cell (17). In that work, we extensively investigated the *Haloarcula hispanica* CreTA, which consists of two RNA components, CreT and CreA. CreT is a small toxic RNA that carries a four-codon open reading frame (ORF) with two consecutive minor arginine codons (AGA) and arrests cellular growth by sequestering the cognate, rare transfer RNA (tRNA^Arg^_UCU_). *H. hispanica* CreA is a variant of crRNA that lacks the canonical 3′ handle (type I crRNAs typically carry 5′ and 3′ flanking handles) and directs Cascade to suppress toxin expression based on its partial complementarity to the promoter of the toxin gene. These insights into the modulation of *H. hispanica* CreTA by CRISPR-Cas are critical for our understanding of the multifunctionality of CRISPR-Cas and its evolutionary and functional entanglement with toxin-antitoxin (TA) modules. However, because both the toxin and the antitoxin components are small RNAs, CreTA modules are extremely diversified and poorly conserved in sequence, which largely impedes the systematic bioinformatic analysis of their distribution and, particularly, the homology-based prediction of their toxin genes.

Here, we experimentally characterize another CreTA module that frequently associates with the type I-B CRISPR-Cas in *Halobacterium hubeiense* strains, and uncover that *H. hubeiense* CreA is structurally more closely similar to a canonical crRNA than the *H. hispanica* CreA and carries two flanking repeat-derived handles. However, the flanking repeats of *creA* are highly divergent and degenerated in sequence, which hindered their interpretation as a minimal CRISPR array. Interestingly, we found that two highly divergent, degenerated repeats seem to be a common feature for most archaeal and bacterial *creA* genes, suggesting their origin and degeneration from a minimal CRISPR structure. By dissecting the elements and mechanism of *H. hubeiense* CreT, we also uncover a group of minimal RNA toxins that specifically sequester the rare isoleucine tRNA. By comparative analysis of *H. hubeiense* and *H. hispanica* CreT toxins, we characterize their convergent strategy to sequester a specific rare tRNA and their dependence on efficient translation signals. These data provide essential information on the origin of CreTA and for future CreTA prediction, and offers more insights into the cryptic small RNAs that associate and co-evolve with CRISPR-Cas.

## MATERIALS AND METHODS

### Strains and growth conditions

*H. hispanica* strains (derivatives of *H. hispanica* ATCC 33960 Δ*pyrF* strain DF60 (18)) used in this study (Supplementary Table S1) were cultivated at 37°C in AS-168 medium (per liter, 200 g NaCl, 20 g MgSO_4_·7H_2_O, 3 g trisodium citrate, 2 g KCl, 50 mg FeSO_4_·7H_2_O, 0.36 mg MnCl_2_·4H_2_O, 5 g Bacto Casamino Acids, 5 g yeast extract, 1 g sodium glutamate, pH was adjusted to 7.2 with sodium hydroxide) and uracil was added to a concentration of 50 mg/l. The strains carrying the pWL502 derivatives were grown in the modified AS-168 medium without yeast extract. Agmatine was added to a final concentration of 570 mg/l, when specified.

*Escherichia coli* JM109 was cultivated at 37°C in Luria-Bertani medium and used as host strain for plasmid engineering. When needed, ampicillin was supplemented to a final concentration of 100 mg/l.

### Plasmid construction and transformation

**Figure 1.**
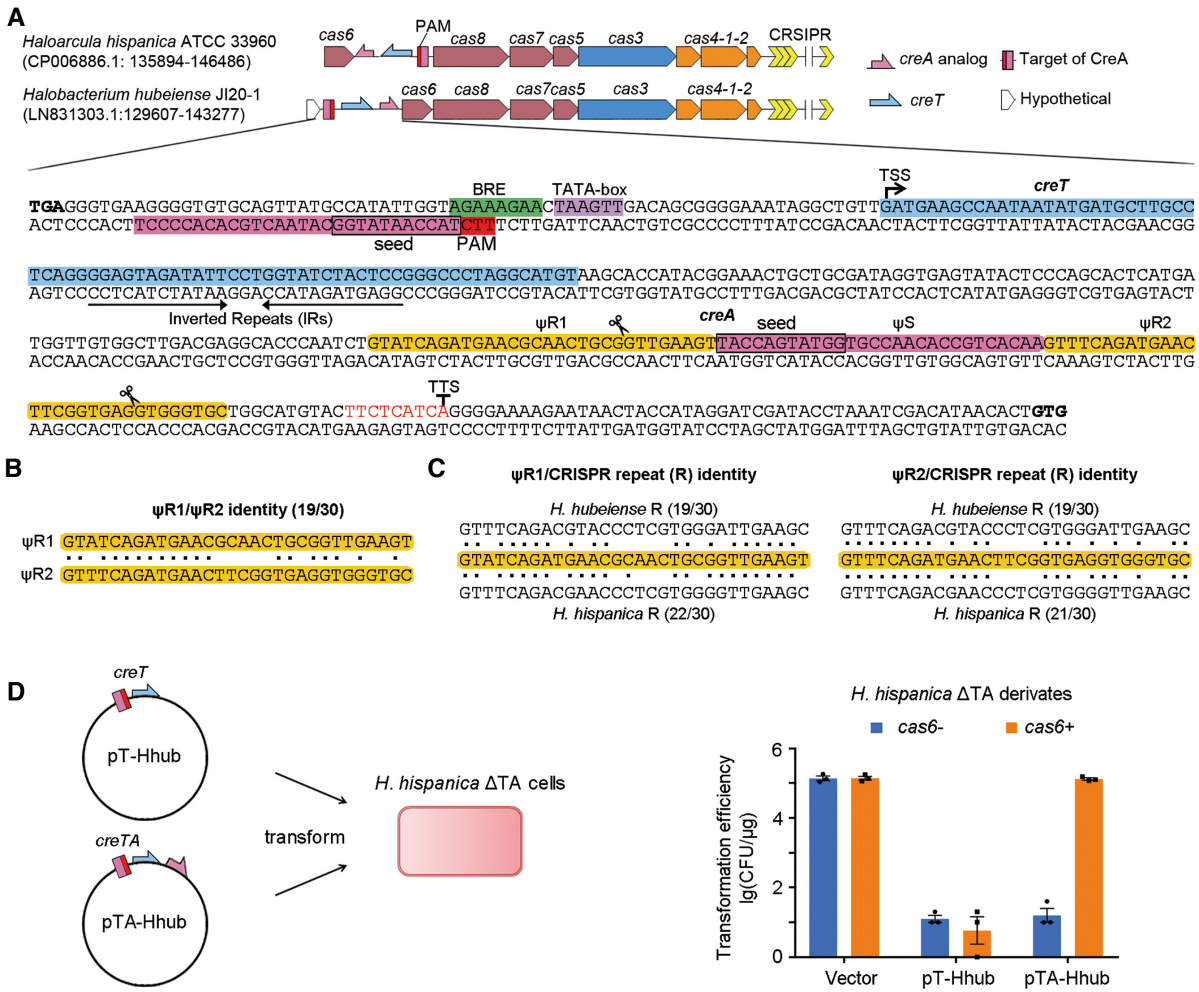
*H. hubeiense creTA* is heterologously regulated by *H. hispanica* CRISPR-Cas. (**A**) The *H. hubeiense creTA* operon (the *H. hispanica creTA* is depicted for comparison). Promoter elements (BRE and TATA-box), transcription start site (TSS) and termination site (TTS) are indicated, respectively. *H. hubeiense creA* consists of two CRISPR repeat analogs (ψR1 and ψR2) sandwiching a spacer analog (ψS). Scissors indicate processing of *creA* transcripts by Cas6. PAM, protospacer adjacent motif. (**B**) The sequence identity between ψR1 and ψR2. (**C**) The sequence identity between a ψR sequence (ψR1 or ψR2) and the *H. hubeiense* (above ψR sequences) or *H. hispanica* (below ψR sequences) CRISPR repeat. (**D**) Transformation of *H. hispanica* cells with a plasmid expressing *H. hubeiense creT* or *creTA*. Error bars, mean ± s.d. (*n* = 3). Scattered dots indicate individual values.

The primers that were used in this study are listed in Supplementary Table S2. The *H. hubeiense* CreTA locus (see Figure 1A for sequence) was commercially synthesized (GenScript, Nanjing, China). Diverse truncated versions of CreTA were amplified from the synthetic DNA template using the high-fidelity KOD-Plus DNA polymerase (TOYOBO, Osaka, Japan). The double-stranded DNA fragments were digested with BamHI and KpnI (New England Biolabs, MA, USA), and ligated into the predigested pWL502 (19) backbone. Overlap extension polymerase chain reaction (PCR) was performed to introduce point mutations as previously described (17). Plasmids were validated by DNA sequencing and subsequently introduced into the *H. hispanica* cells according to the online Halohandbook (https:// haloarchaea.com/wp-content/uploads/2018/10/Halohandbook_2009_v7.3mds.pdf). The yeast extract-subtracted AS-168 was used as the selective medium. The log-transformed data of transformation efficiency (CFU/μg) were used to calculate the average and the standard deviation, and also to perform the two-tailed Student's *t* test.

### RNA extraction and RNA-seq analysis

The *H. hispanica* cells encoding (*cas6+*) or lacking (*cas6-*) Cas6 were transformed with pTA-tRNA. Colonies were randomly selected, separately inoculated into 10 ml of yeast extract-subtracted AS-168 medium (containing 570 mg/l agmatine), and then cultured for 4 days. After sub-inoculation and another 2-day culturing, the exponential-phase cells were collected by centrifugation and the total RNA was extracted using the TRIzol reagent (Thermo Fisher Scientific, MA, USA) according to the standard guidelines. RNA concentration was determined using a Nanodrop 1000 spectrophotometer (Thermo Fisher Scientific, MA, USA). A total of 50 μg of RNA was successively treated with RNA pyrophosphohydrolase and T4 polynucleotide kinase [both purchased from New England Biolabs (MA, USA)] according to the manufacturer's protocols. The pretreated RNA was purified using the phenol:chloroform method, followed by precipitation with the same volume of isopropanol and 0.1 volume of 3 M sodium acetate. The RNA quality was analyzed using Nanodrop 2000 (Thermo Fisher Scientific, MA, USA) prior to constructing the RNA-Seq library. Small RNA libraries were constructed with RNA molecules ranging from 30 to 300 nt, following the guideline of the NEXTFLEX Small RNA-Seq Kit (Bioo Scientific, TX, USA), and then subjected to 150-bp paired-end sequencing on an Illumina HiSeq X Ten. The raw reads were trimmed to remove adapters and low-quality reads. A custom Perl script was finally used to map the resulting reads to the *creTA* sequence (17).

### Northern blot analysis

The early-stationary culture of random-selected colonies was sampled and total RNA was extracted using the TRIzol reagent. A total of 10 μg of RNA was denatured at 65°C for 10 min with equal volume of RNA loading dye (Takara, Shiga, Japan). RNA samples, the Century-Plus RNA ladder (Thermo Fisher Scientific, MA, USA), and the biotin-labeled single-stranded DNA (serving as a custom size marker) were separated on an 8% polyacrylamide gel (7.6 M urea). Electrophoresis was performed in 1× TBE buffer at 200 V for approximately 1 h. The lane of the commercial RNA ladder was excised, stained by ethidium bromide, and then imaged. The remnant RNA samples and custom markers were transferred onto Biodyne B nylon membrane (Pall, NY, USA) by electroblotting and then UV-crosslinked to the membrane. The membrane was hybridized with a biotin-labeled probe for approximately 12 h, and the signals were visualized using the Chemiluminescent Nucleic Acid Detection Module Kit (Thermo Fisher Scientific, MA, USA) according to the manufacturer's protocol.

### Fluorescence measurement

The gene of a soluble-modified red-shifted GFP protein (20) was used to report the activity of P*_creTA_*. For each *H. hispanica* transformant (with the *gfp*-carrying plasmids or the empty vector), three individual colonies were randomly selected and separately inoculated with 10 ml of yeast extract-subtracted AS-168 medium. After cultivation to the late exponential phase, the cell culture was sampled and fluorescence was measured. Fluorescence intensity and OD_600_ were simultaneously determined using a microplate reader (BioTeck, VT, USA), and their ratio was used for plotting.

### Bioinformatic analysis

The protein-coding genes of *H. hispanica* were downloaded from NCBI (https://ftp.ncbi.nlm.nih.gov/genomes/all/GCA/000/223/905/GCA_000223905.1_ASM22390v1/) and the usage frequency of Ile codons was calculated. RNAfold webserver (21) was used to analyze the folding potential of CreT RNA. Sequence alignments were constructed using the T-Coffee webserver, the homology of sequences was analyzed by the GeneDoc software (version 2.6.002).

## RESULTS

### *H. hubeiense creTA* and its regulation by *H. hispanica* CRISPR-Cas

Different from the *H. hispanica creTA* that resides within the intergenic region between *cas6* and *cas8*, *H. hubeiense creTA* is located immediately upstream of the *cas* operon (Figure 1A). As in the case of *H. hispanica*, *H. hubeiense creA* contains a CRISPR spacer-like (ΨS) sequence, of which the first 1–5 and 7–11 nucleotides base pair to a DNA sequence upstream of *creT*, and notably, the target sequence (protospacer) is flanked by 5′-TTC-3′, which is the PAM motif of type I-B CRISPR-Cas (12,22). In *H. hispanica*, ΨS is preceded by a CRISPR repeat-like sequence (ΨR) and ends with a thymine-rich transcription terminator (17). By contrast, the *H. hubeiense* ΨS appears to be sandwiched by two CRISPR repeat-like sequences (30 nt each), denoted as ΨR1 and ΨR2, respectively (Figure 1A). Notably, ΨR1 and ΨR2 share only 19 identical nucleotides (Figure 1B), which hindered their discovery and interpretation as a minimal CRISPR array. Interestingly, compared to ΨR2, the last 8 nucleotides of ΨR1 are more similar to those of the canonical CRISPR repeat (Figure 1C), which are transcribed into the 5′ handle of mature crRNAs (10).

Because the transformation method has not been established for *H. hubeiense*, in our previous study, we cloned its putative *creT* gene into the pWL502 vector, and found that the recombinant plasmid (named pT-Hhub) transformed *H. hispanica* cells with a markedly reduced efficiency (∼10^4^-fold) compared to the empty vector (17), indicating that the *H. hubeiense* CreT is functional and toxic in *H. hispanica*. Then, we noticed that both ΨR1 and ΨR2 share more sequence similarity to the *H. hispanica* CRISPR repeat than to the *H. hubeiense* CRISPR repeat (Figure 1C), leading to the prediction that *H. hubeiense* CreA also would be functional in *H. hispanica*. We cloned the *H. hubeiense creTA* operon into the vector (pTA-Hhub), and transformed *H. hispanicacas6+ and cas6-* strains (note that the native *creTA* was pre-deleted from both strains) (Figure 1D). We found that pTA-Hhub transformed *cas6+* cells with a high efficiency comparable to the empty vector (∼10^5^ CFU/μg; CFU, colony-forming unit), but transformed *cas6-* cells with a markedly reduced efficiency (∼10^4^-fold). By contrast, pT-Hhub that carries only the *creT* gene showed very low efficiency (∼10 CFU/μg) in transforming both *cas6+* and *cas6-* cells (Figure 1D). It was indicated that *H. hubeiense creA* suppressed its cognate *creT*, jointly with *H. hispanica* Cas6 and probably other Cas proteins. Then, we tested each *cas* mutant of *H. hispanica*. As expected, pTA-Hhub caused toxicity (∼10^4^-fold reduction in transformation efficiency compared to the empty vector) in cells lacking any or all of the *cascade* genes, but not in those lacking *cas1*, *cas2*, *cas3* or *cas4* (Supplementary Figure S1). We concluded that the activity of the heterologous *creTA* from *H. hubeiense* was modulated by the *H. hispanica* CRISPR effector.

### *H. hubeiense* CreA closely resembles crRNA and contains two Cas6-processed handles

**Figure 2.**
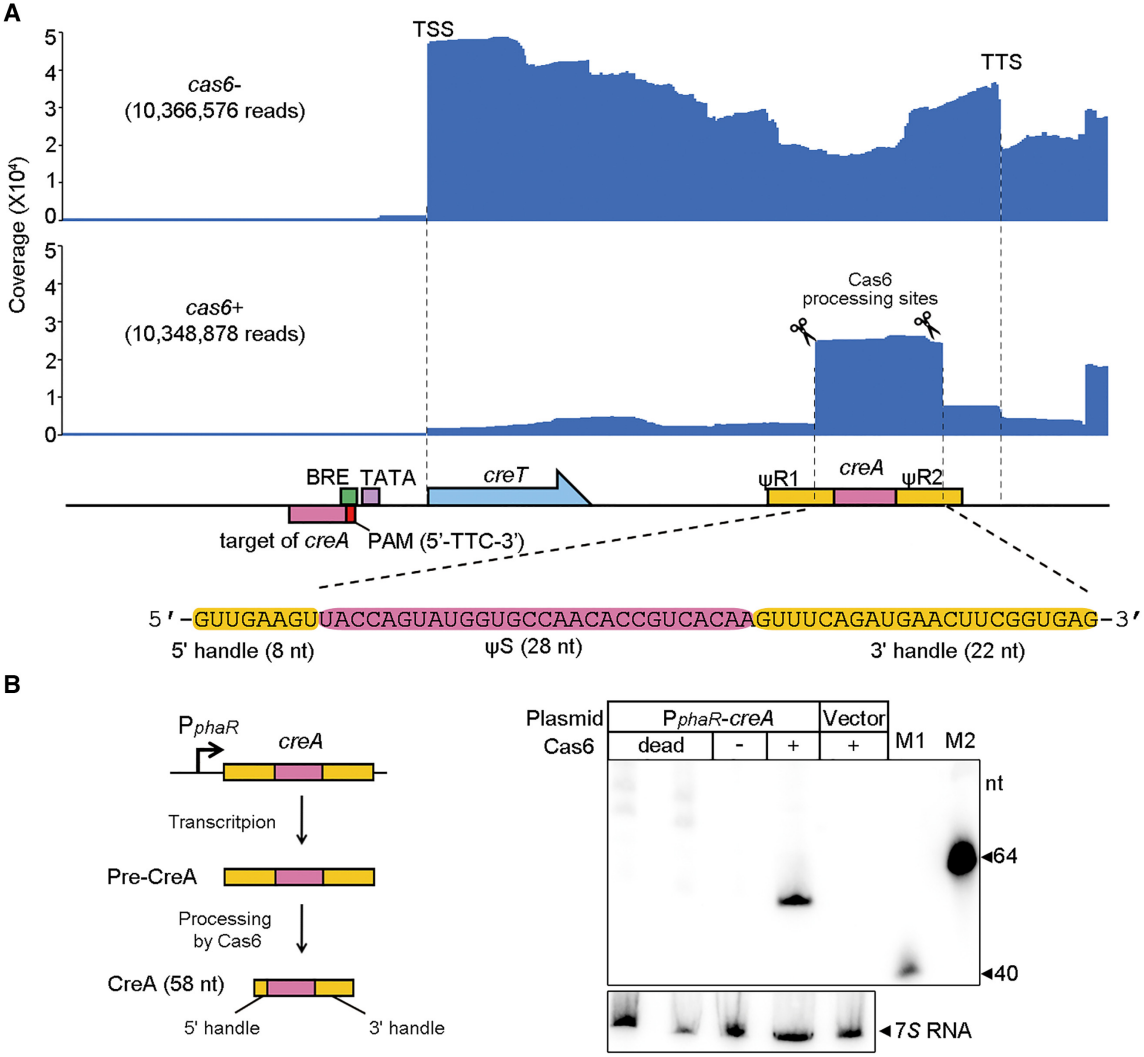
*H. hubeiense* CreA resembles a typical two-handle crRNA. (**A**) Transcription profile of *creTA* operon in the presence or the absence of *H. hispanica cas6*. Note that pTA-tRNA was used to overexpress a tRNA (see Figure 5D) to relieve the toxicity of *creT* in *cas6-* cells. (**B**) Northern blotting of *H. hubeiense* CreA in *H. hispanica* cells lacking Cas6 (-) and in those encoding a wild-type (+) or a catalytically-dead Cas6 (in duplicate). A strong promoter (P*_phaR_*) was used to drive the transcription of *H. hubeiense creA*. M1 and M2, biotin-labeled 40-nt and 64-nt ssDNA, respectively.

We explored the transcription profile of *H. hubeiense creTA* in the *H. hispanica cas6+* or *cas6-* cells using small RNA sequencing (sRNA-seq) (Figure 2A). For this assay, we used a pTA-Hhub derivative co-expressing tRNA^Ile^_CAU_, which could relieve the toxicity of CreT in *cas6-* cells (see below). Furthermore, the RNA samples were pretreated by polynucleotide kinase and 5′ pyrophosphohydrolase to activate the 5′ terminus of mature CreA (hydroxylated) and that of nascent *creTA* transcripts (triphosphorylated), respectively (see Materials and Methods). Sequencing of the *cas6-* RNA samples revealed the strong transcription start site (TSS) of the *creTA* operon (Figure 2A), upstream of which we predicted the promoter elements BRE (TF-IIB recognition) and TATA-box (23) (Figure 1A). Driven by this promoter (hereafter P*_creTA_*), abundant transcripts extended and gradually decreased along the *creTA* operon, with a prominent transcription termination site (TTS) appearing downstream of *creA* (Figure 2A). Interestingly, a fraction of transcripts started within ΨR2, suggesting ΨR1 and ΨS contain sequences that promoted accidental transcription (or perhaps ΨR2 contains the cutting site of unknown ribonucleases). sRNA-seq of the *cas6+*samples revealed the extensive accumulation of mature CreA RNA, which was not observed in the *cas6-* samples (Figure 2A). Notably, the mature CreA carries an 8-nt 5′ handle and a 22-nt 3′ handle (Figure 2A), which are the typical feature of a canonical crRNA (7,24). We hypothesized that, although the two repeat-like units ΨR1 and ΨR2 are highly divergent in sequence, they were both recognized and processed by *H. hispanica* Cas6. By Northern blotting, we examined CreA RNA in *H. hispanica* cells expressing a catalytically dead Cas6 (H37A mutant). Although a constitutive promoter (P*_phaR_*) (25) was employed to control the expression of *H. hubeiense creA*, mature CreA RNA was detected only in cells encoding the wild-type Cas6, but not in cells lacking Cas6 or expressing its catalytically dead mutant (Figure 2B). Therefore, maturation of *H. hubeiense* CreA depends on the nucleolytic activity of Cas6 on the two highly divergent flanking ‘repeats’.

### Two-ΨR *creA* is more common among CreTA modules

**Figure 3.**
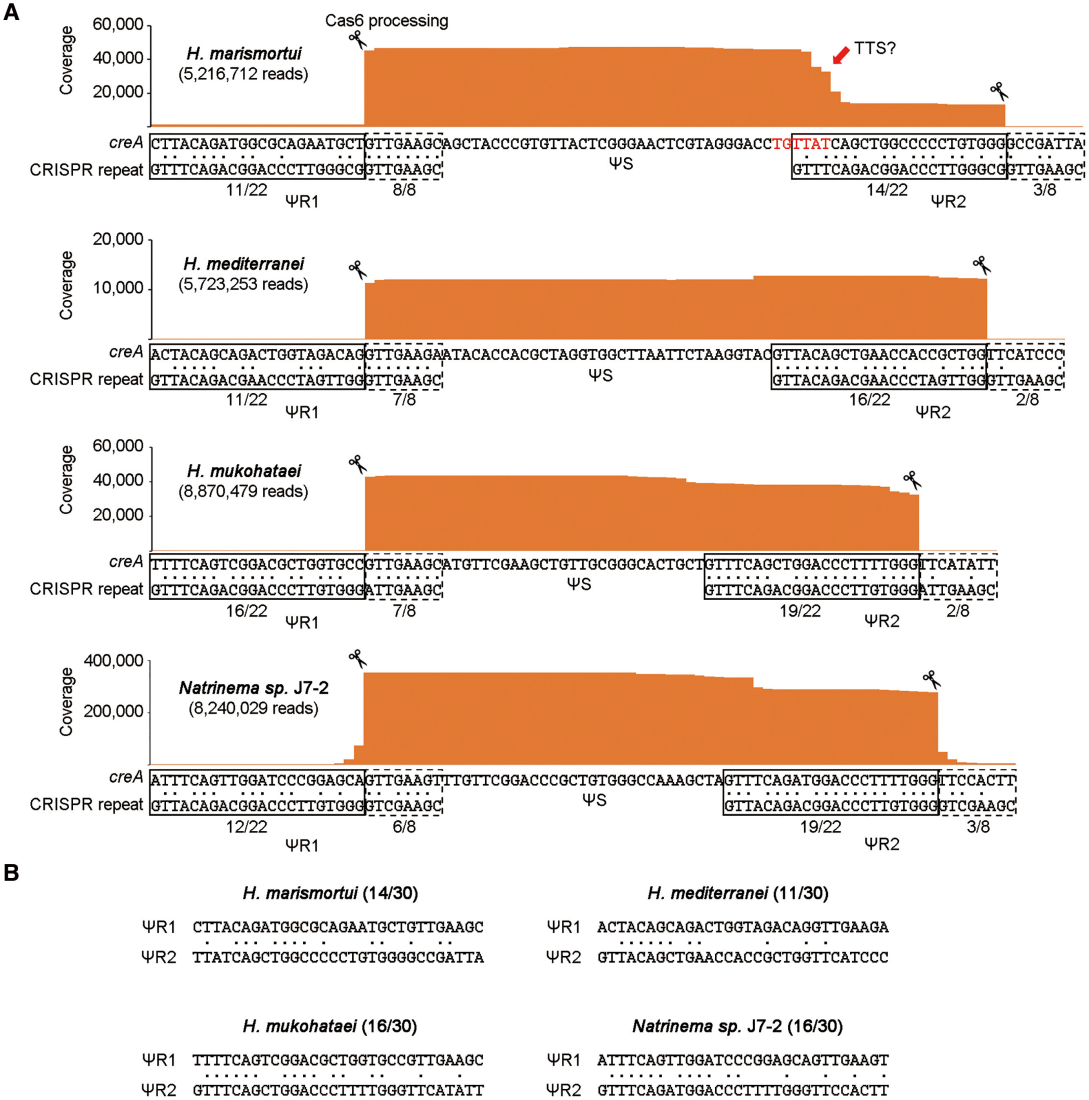
Another four haloarchaeal *creA* genes with two highly divergent, degenerated flanking ‘repeats’ (ΨR sequences). (**A**) Analysis of the *creA* RNA products by sequencing the small RNA from *Haloarcula marismortui* ATCC 43049, *Haloferax mediterranei* ATCC 33500, *Halomicrobium mukohataei* DSM 12286, and *Natrinema* sp. J7-2. Red nucleotides indicate a putative transcription terminator (TTS, transcription termination site). Identity between *creA* ΨR and the co-occurring CRISPR repeat was calculated separately for the first 22 nucleotides and the last eight nucleotides. (**B**) Sequence identity between ΨR1 and ΨR2.

Knowing that the *H. hubeiense creA* gene has a second CRISPR repeat-like sequence (ΨR2) that was significantly different from the first one (ΨR1) but also processed by the Cas6 nuclease, we wondered whether more *creTA* modules actually also carry two highly divergent flanking ‘repeats’, instead of one. We reanalyzed the sequence of another four haloarchaeal *creTA* modules predicted in our previous study (17), and found that each *creA* gene does contain two ΨR sequences (Figure 3A), which, however, share very limited sequence identity with each other (ranging from 11/30 to 16/30) (Figure 3B). Then we extracted RNA samples from these four haloarchaeal strains and performed small RNA sequencing. The sRNA-seq data demonstrated that the two ΨR sequences of each *creA* gene could generate an 8-nt 5′ handle and a 22-nt 3′ handle, respectively (Figure 3A), and hence their mature CreA RNAs consistently have the typical architecture of crRNAs. Interestingly, the *Haloarcula marismortui creA* gene also produced a large fraction of smaller RNAs which carry only the 5′ handle with a 3′ terminus that possibly derived from transcription termination at the beginning of ΨR2 (Figure 3A). The architecture of this one-handle CreA is reminiscent of the RNA products from the one-ΨR *creA* gene of *H. hispanica* (17). It is possible that the one-handle and the two-handle RNA products of *H. marismortui creA* are both functional as two isoforms.

Our previous study also predicted several bacterial CreTA modules (17). We further reanalyzed their sequences and found that their *creA* genes also each carry two CRISPR repeat-like sequences, which, again, share very limited sequence identity with each other (Supplementary Figure S2). Therefore, we conclude that it is a common feature of archaeal and bacterial *creA* genes to carry two highly divergent, degenerated flanking repeats, which, however, has retained the critical nucleotides for Cas6 recognition and processing. Accordingly, the initially characterized one-ΨR *creA* from *H. hispanica* may have lost the second ΨR as a transcription terminator evolved to generate a more concise form of antitoxin RNA (like the smaller RNA produced from *H. marismortui creA*).

### *H. hubeiense* CreA represses P*_creTA_* based on their partial complementarity

From the sRNA-seq results of *H. hubeiense creTA*, the abundant *creT* transcripts observed in the *cas6-* samples dramatically decreased (by >30-fold) in the *cas6+* samples (Figure 2A), suggesting the operon promoter P*_creTA_*was efficiently auto-repressed by mature CreA. To confirm this regulatory effect, we replaced the *creT* gene on pT-Hhub and pTA-Hhub with a gene of green fluorescent protein (*gfp*), generating pT-gfp and pTA-gfp, and monitored their fluorescence in *H. hispanica* cells (Supplementary Figure S3). pT-gfp (lacking *creA*) produced fluorescence of equivalent intensity in *cas6+* and *cas6-* cells. Such intensive fluorescence was also observed for pTA-gfp (carrying *creA*) in *cas6-* cells, but in *cas6+* cells (where CreA was matured by Cas6), fluorescence decreased by ∼26-fold. We then introduced pT-gfp and pTA-gfp separately into cells lacking *cas1*, *cas2*, *cas3*, *cas4* or other *cascade* genes, and monitored their fluorescence. The fluorescence from pT-gfp was higher than from pTA-gfp in cells lacking *cas1*, *cas2*, *cas3* or *cas4*, and by contrast, equivalent fluorescence was produced from the two plasmids in cells lacking one or all of the *cascade* genes (Supplementary Figure S4). These results substantially support that CreA auto-regulates P*_creTA_* jointly with Cascade.

**Figure 4.**
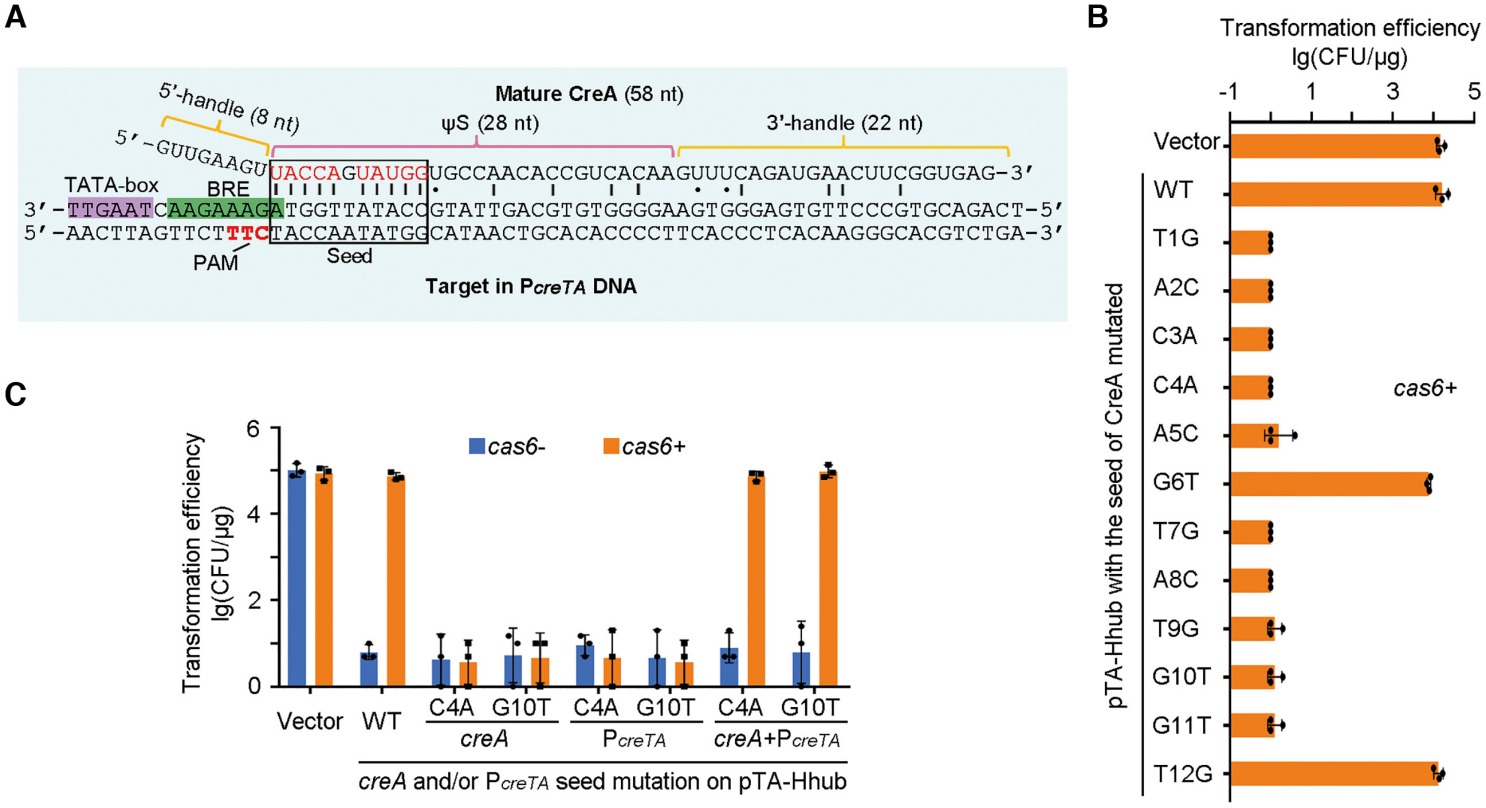
The partial complementarity between CreA and P*_creTA_* is critical for toxin repression. (**A**) Mature CreA RNA and its complementarity to P*_creTA_*. ‘|’ and ‘•’ indicate Watson-Crick and wobble base pairings, respectively. Red nucleotides in the seed region form critical base pairings to P*_creTA_*. (**B**) Single mutation of the seed nucleotides of *creA*. (**C**) Complementary mutation of *creA* and P*_creTA_*. The 4th (or 10th) nucleotide in the spacer of *creA* and that in the protospacer of P*_creTA_* were separately or simultaneously mutated. *H. hispanica* cells encoding (*cas6*+) or lacking (*cas6*-) Cas6 were transformed with pTA-Hhub derivates. Error bars, mean ± s.d. (*n* = 3). Scattered dots indicate individual values.

This regulation is in accord with the observation that the first 1–5 and 7–11 nucleotides (seed) of CreA ΨS base pair to the identified P*_creTA_*, with the PAM sequence 5′-TTC-3′ located within the complement of the purine-rich BRE element (Figure 4A). We constructed a series of pTA-Hhub derivatives by mutating each of the first 12 nucleotides of ΨS (Figure 4B). When the 6th or 12th nucleotide (not participating in crRNA-target DNA base pairing (26)) was mutated, the *cas6+* cells were transformed with an efficiency comparable to that of the empty vector and the WT pTA-Hhub. By contrast, when any of the other 10 nucleotides was altered, a ∼10^4^-fold reduction in transformation efficiency was observed (Figure 4B). We therefore hypothesized that these 10 seed nucleotides (1–5 and 7–11; red colored in Figure 4A) form the minimal complement to P*_creT_* that is required for the antitoxic role of CreA. Consistently, when we modified P*_creT_* to interrupt this complementarity (C4A and G10T; Figure 4C), the WT *creA* no longer suppressed *creT* and the mutated plasmid showed minimal transformation efficiency (∼10 CFU/μg) in both *cas6-* and *cas6+*cells. By contrast, the complementarily mutated *creA* restored high transformation efficiency (∼10^5^ CFU/μg). Therefore, the regulatory role of CreA depends on its limited but critical ‘seed complementarity’ to P*_creTA_*. Interestingly, *H. hispanica* CreTA (17) and *H. hubeiense* CreTA evolved exactly the same seed complementarity (at nucleotides 1–5 and 7–11) to achieve CreA-guided transcriptional regulation of *creT*.

### *H. hubeiense* CreT acts by sequestering the rare tRNA^Ile^_CAU_

**Figure 5.**
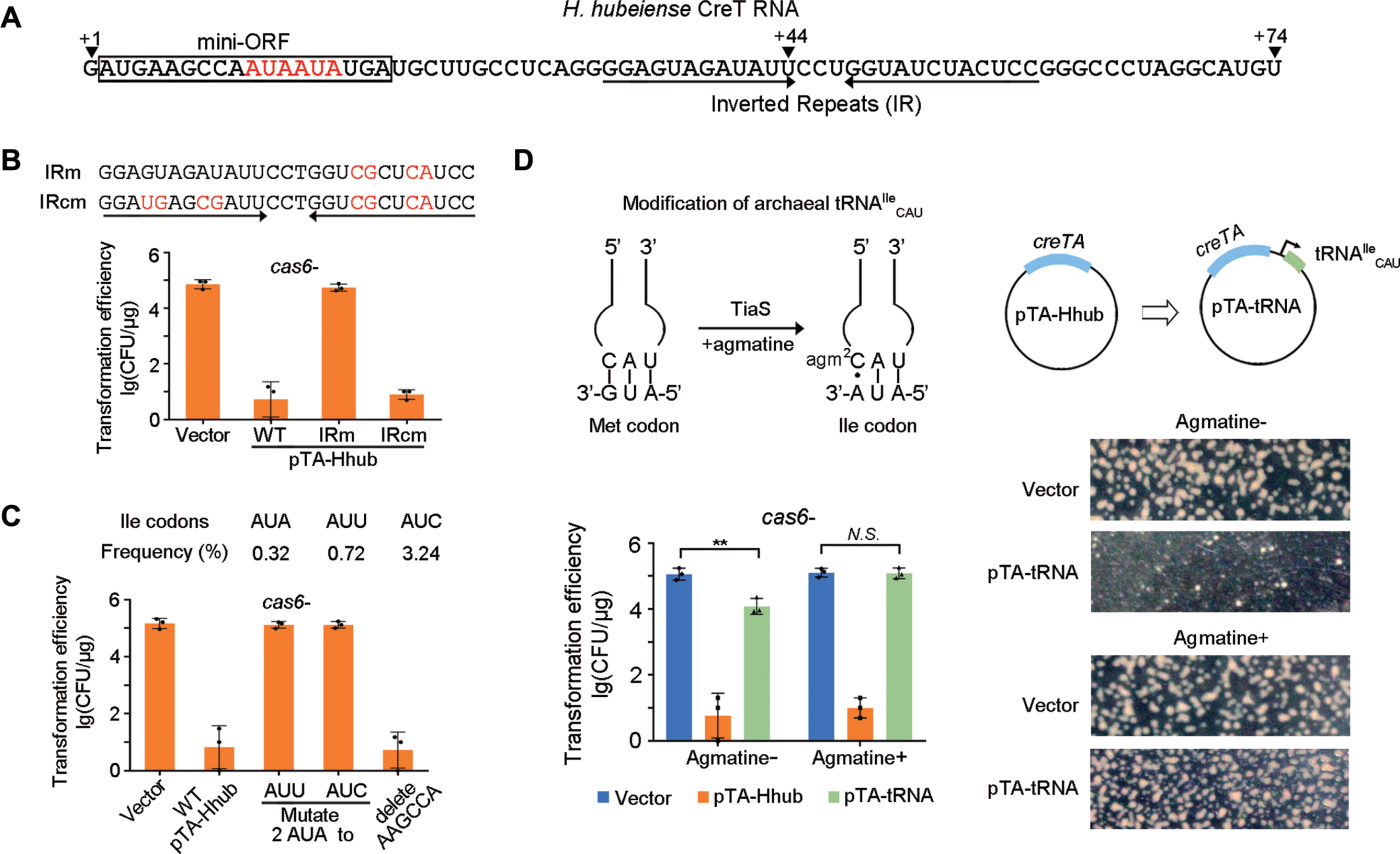
*H. hubeiense* CreT acts by sequestering the AUA-decoding tRNA^Ile^_CAU_. (**A**) Sequence and elements of *H. hubeiense* CreT RNA. ‘+1’ indicates the TSS, while ‘+44’ and ‘+74’ indicate two very weak TTSs. The mini open reading frame (ORF) contains two consecutive AUA codons (highlighted in red). (**B**) CreT toxicity when the complementarity between the inverted repeats was disrupted and then recovered. (**C**) Synonymous mutation of the two AUA codons and deletion of the two preceding sense codons (AAG and CCA). The usage frequency of each isoleucine codon (among all codons) in *H. hispanica* is given. (**D**) Suppression of *H. hubeiense* CreT by over-expressing tRNA^Ile^_CAU_ and/or supplementing agmatine. Representative images of the transformant colonies are given. Error bars, mean ± s.d. (*n* = 3); two-tailed Student's *t* test (**P < 0.01, N.S., not significant (*P* > 0.05)). Scattered dots indicate individual values.

Then, we asked how *H. hubeiense* CreT caused toxicity in *H. hispanica* cells. Our previous study on *H. hispanica* CreT demonstrated that this RNA toxin acts by sequestering tRNA^Arg^_UCU_ that deciphers the rare AGA codons (17). A strong Shine-Dalgarno motif, an efficient start codon (AUG or GUG), two consecutive AGA codons located immediately downstream, and a stable stem−loop structure are all critical for its activity. Combination of these elements was not found in the sequence of *H. hubeiense* CreT. However, we noticed a pair of inverted repeats (12 nt each) that have the potential to fold into a stable stem-loop structure (Figure 5A). We truncated pT-Hhub to eliminate one of the two inverted repeats, and found that CreT no longer caused toxicity, and as a result, the plasmid transformed *H. hispanica* cells rather efficiently (>10^4^ CFU/μg) (Supplementary Figure S5). Similarly, when the repeat was mutated to disrupt the folding potential, toxicity was not observed either (IRm in Figure 5B). When the other repeat was further mutated to restore the folding potential, CreT became toxic again and the transformation efficiency was markedly reduced (IRcm in Figure 5B). Both the truncation and mutation assays supported the importance of the stem-loop structure for CreT activity. Then we analyzed the sequence upstream of the stem-loop and noticed a six-codon open reading frame (denoted mini-ORF), which, remarkably, contains two consecutive AUA codons (Figure 5A). By analyzing the *H. hispanica* genome, we showed that AUA is least utilized among the three isoleucine codons (AUA, AUU and AUC) (Figure 5C). Assuming that *H. hubeiense* CreT acts in a manner similar to the *H. hispanica* toxin and sequesters the tRNA decoding the rare AUA codons, we replaced these two AUA codons by the more common AUU or AUC isoleucine codons. As expected, the pTA-Hhub derivatives transformed *H. hispanica cas6-* cells with a high efficiency (∼10^5^ CFU/μg) (Figure 5C), suggesting that the synonymous mutations inactivated CreT. Then, we deleted the AAGCCA sequence between the start codon and the rare codons, and found that the mutated CreT remained toxic (Figure 5C). Therefore, like *H. hispanica* CreT, the *H. hubeiense* toxin acts not by encoding a small peptide, but rather by overusing a minor codon (AUA in this case), which can lead to the sequestration of its cognate tRNA.

In fact, AUA is a unique codon that is translated by tRNA^Ile^_CAU_, and this process strictly relies on the modification of the first (wobble) position of the anticodon CAU (27–29). In archaea, the wobble base cytidine (C) of tRNA^Ile^_CAU_ is modified to 2-agmatinylcytidine (agm^2^C) (Figure 5D), which allows it to form two hydrogen bonds with the third base (adenine, A) of AUA codons (27). We modified pTA-Hhub by adding the *H. hispanica* tRNA^Ile^_CAU_ gene (HAH_2749) under the control of a strong promoter, thus generating the plasmid pTA-tRNA (Figure 5D). When transforming *H. hispanica cas6-* cells, pTA-tRNA showed an efficiency that was much higher (∼10^3^-fold) than pTA-Hhub. However, compared to the empty vector (pWL502), there was still a ∼10-fold reduction in transformation efficiency, and notably, its transformants formed much smaller colonies (Figure 5D). Thus, overexpression of tRNA^Ile^_CAU_ only partly relieved the toxicity of *H. hubeiense* CreT. We hypothesized that the over-expressed tRNA^Ile^_CAU_ had not been fully modified due to the scarcity of agmatine supply. Hence, we supplemented agmatine to the medium used for transformant screening. As expected, the transformation efficiency of pTA-tRNA was elevated to the same level as the empty vector, and the transformants formed normal colonies on the agmatine-plus plates (Figure 5D). These data collectively demonstrate that *H. hubeiense* CreT sequesters tRNA^Ile^_CAU_ that decodes the rare AUA codons.

### Engineering *H. hubeiense* CreT to sequester a rare arginine tRNA

**Figure 6.**
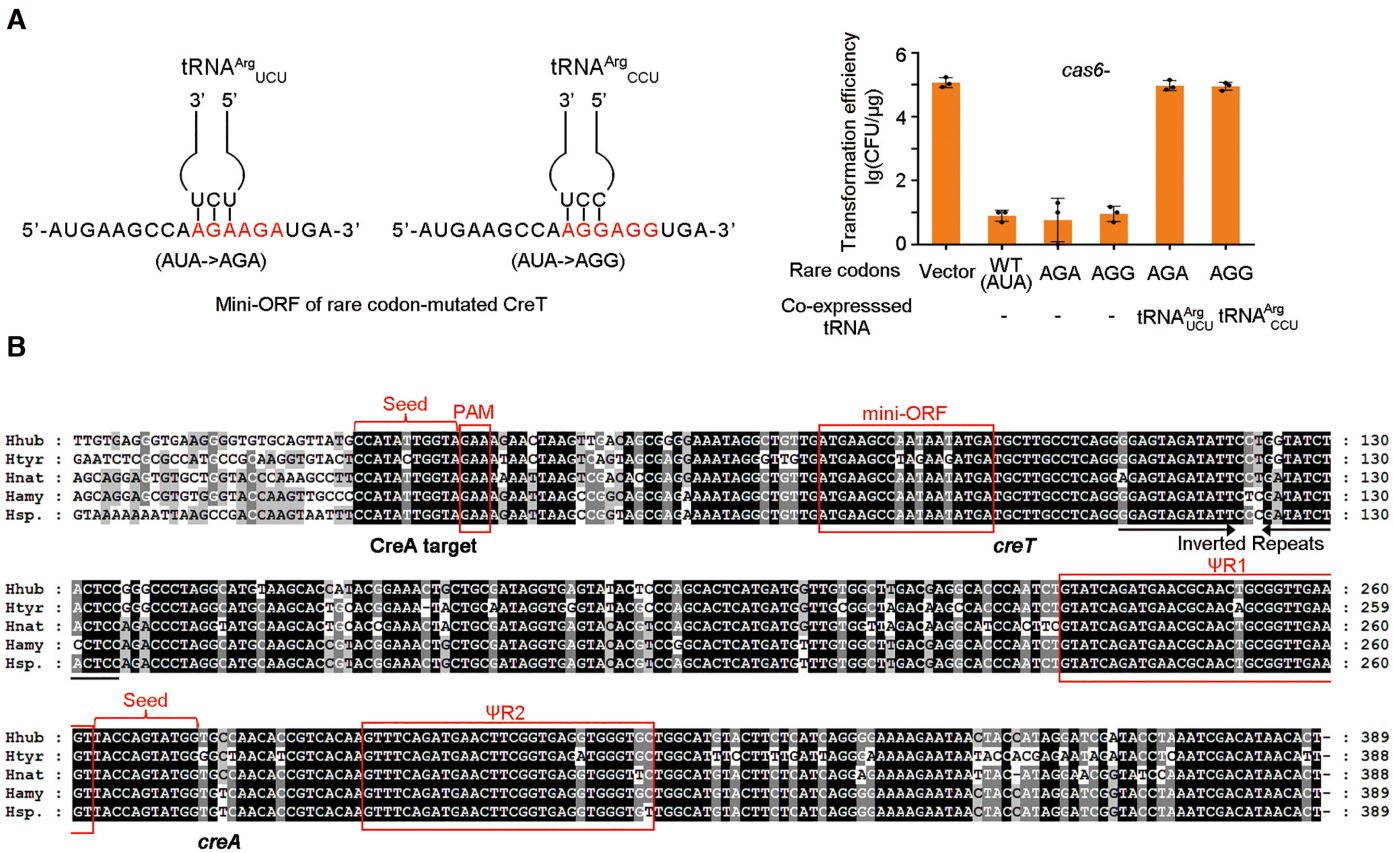
Engineering *H. hubeiense* CreT to sequester rare arginine tRNAs. (**A**) Toxicity of CreT mutants sequestering rare arginine tRNAs and their suppression by over-expressing the corresponding tRNA. Error bars, mean ± s.d. (*n* = 3). Scattered dots indicate individual values. (**B**) Four closely-related homologs of *H. hubeiense* (Hhub) *creTA* overusing rare isoleucine (AUA) or arginine (AGA) codons. Htyr, *Halobonum tyrrellensis* G22; Hnat, *Haloplanus natans* DSM 17983; Hamy, *Halorubrum amylolyticum* strain ZC67; Hsp., *Halorubrum* sp. Atlit-26R.

Although *H. hispanica* and *H. hubeiense* CreT toxins share little sequence similarity, they both arrest cellular growth by sequestering rare tRNA species that decode minor codons. *H. hispanica* CreT sequesters tRNA^Arg^_UCU_ with two consecutive AGA codons, whereas *H. hubeiense* CreT sequesters tRNA^Ile^_CAU_ with two AUA codons. We sought to determine whether *H. hubeiense* CreT could be engineered to sequester the rare arginine tRNA species. We modified pTA-Hhub by replacing its two AUA codons with the rare AGA or AGG arginine codons (Figure 6A). When used to transform *H. hispanica cas6-* cells, these modified plasmids consistently showed a ∼10^4^-fold reduction in efficiency compared to the empty vector. Notably, their transformation efficiency was recovered to the level of the empty vector by over-expressing tRNA^Arg^_UCU_ and tRNA^Arg^_CCU_ that decode AGA and AGG codons, respectively (Figure 6A). By sequence similarity search of the National Center for Biotechnology Information (NCBI) nucleotide genomic database, we discovered a closely-related homolog of *H. hubeiense* CreT in *Halobonum tyrrellensis* G22, which contains two consecutive AGA instead of AUA codons in the mini-ORF (Figure 6B). Therefore, to arrest cellular growth by sequestering a rare arginine or isoleucine tRNA seems to be a convergently evolved strategy of these small RNA toxins.

### tRNA-sequestering effect depends on translation efficiency of CreT

It could be expected that efficient translation of the mini-ORF containing minor arginine or isoleucine codons should be important for CreT toxicity. Our previous study showed that *H. hispanica* CreT has a strong SD motif (may enhance translation initiation) that is critical for its toxicity (17). By contrast, the *H. hubeiense* CreT is ‘leader-less’ and lacks an SD motif (Supplementary Figure S6A). It seems that, without an SD sequence, *H. hubeiense* CreT can initiate translation efficiently enough to sequester tRNA^Ile^_CAU_. We subjected the start AUG codon of the mini-ORF to saturation mutagenesis. When AUG was mutated to any other triplets, including the two less efficient start codons, GUG and UUG (30), the mutated pTA-Hhub transformed *H. hispanica cas6-* cells with an efficiency comparable to the empty vector (Supplementary Figure S6B), suggesting the CreT mutants were (partly) inactivated. However, we noticed that the transformants of the GUG mutant hardly grew in liquid medium (Supplementary Figure S6C), indicating this mutant was still toxic although apparently less so than the WT. Then, we engineered the GUG and UUG mutants by adding the SD motif from *H. hispanica*, which fully restored their toxicity and resulted in a ∼10^4^-fold reduction in transformation efficiency (Supplementary Figure S6D). We concluded that efficient translation initiation is critical for CreT toxicity, and specifically, the most efficient start codon AUG is crucial for the CreT activity in the absence of a strong SD sequence, whereas in its presence, a less efficient start codon (GUG or UUG) could also initiate a sufficient rate of translation to sequester tRNA^Ile^_CAU_ and arrest cellular growth.

## DISCUSSION

Our recent study unearthed a diverse set of CRISPR-regulated toxin−antitoxin (CreTA) RNA pairs, which safeguard the genetic integrity of the multi-subunit CRISPR effector (mainly type I) and hence protect the adaptive immunity (17). This protective role of CreTA counteracts the fitness costs that CRISPR-Cas imparts on the host cell and prevents elimination of CRISPR by purifying selection. Conceivably, toxin−antitoxin modules associated with CRISPR-Cas and resembling CreTA, at least in terms of the general mechanism of action, could be more common in CRISPR-Cas systems than currently appreciated. Exploring and dissecting the general rules and the diverse mechanisms adopted by such protective CreTA-like systems could substantially contribute to the understanding of CRISPR evolution and functions. Furthermore, exploration of the CRISPR-mediated regulation of CreTA can produce insights into the multifunctionality of Cas proteins.

**Figure 7.**
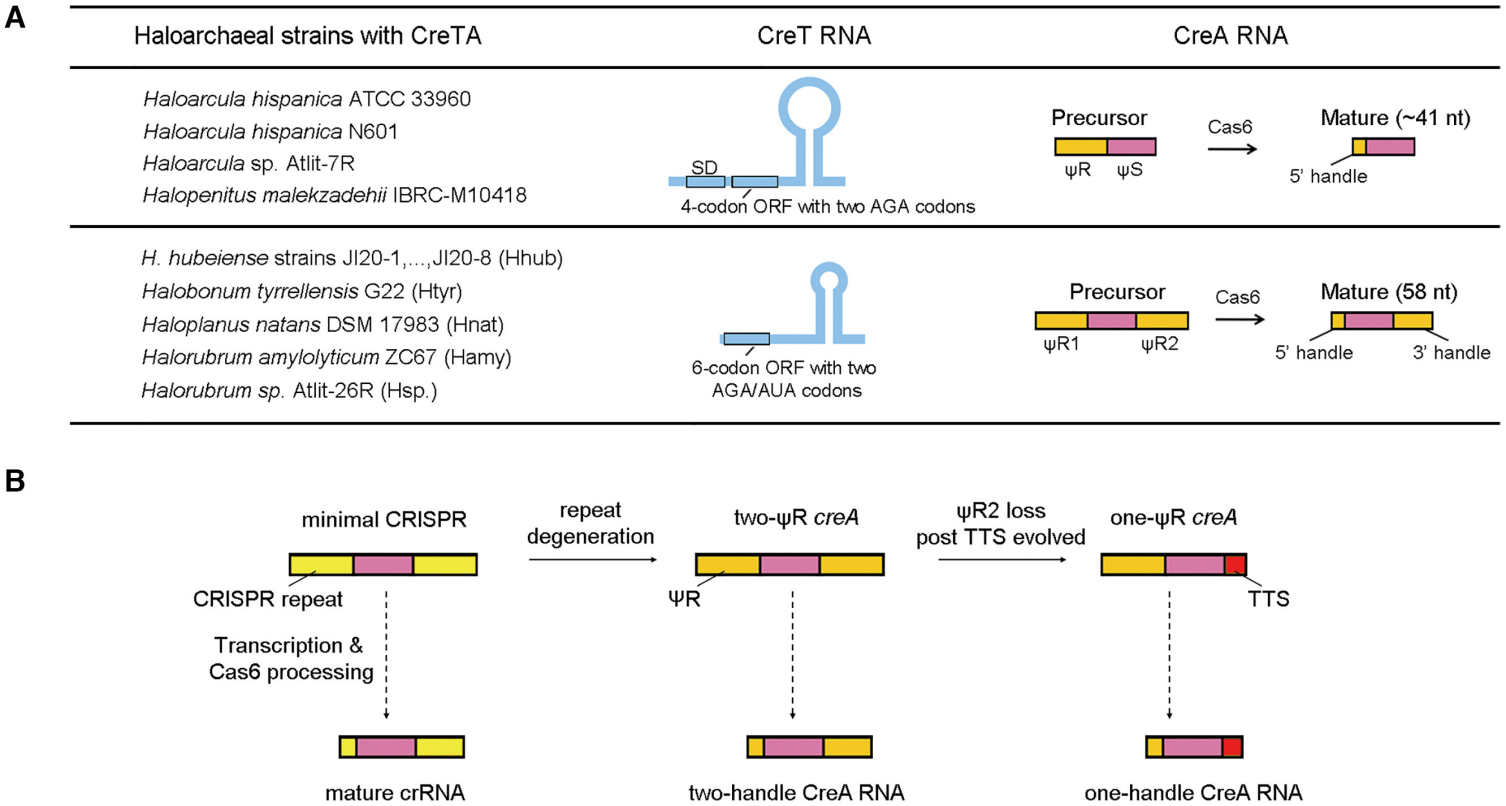
Summary of the two groups of haloarchaeal CreTA modules that sequester the rare arginine (AGA) or isoleucine (AUA) tRNA (**A**) and the origination of two-ΨR and one-ΨR *creA* genes from minimal CRISPRs (**B**). The *creTA* loci in 8 *H. hubeiense* strains (JI20-1 to JI20-8) are identical in sequence (see Figure 6B). SD, Shine-Dalgarno sequence. TTS, transcription termination site.

Because both the toxin and the antitoxin components of CreTA-like systems are small RNAs that are poorly conserved at the sequence level, systematic discovery and mechanistic prediction for such elements is a major challenge (17). We previously dissected the initially discovered CreTA, which is carried and modulated by the *H. hispanica* type I-B CRISPR-Cas (17). In this work, we mainly investigated another CreTA from *H. hubeiense* and characterized its heterologous regulation by the *H. hispanica* CRISPR-Cas. Although these two CreTA modules can plug into the same CRISPR-Cas system and both act by sequestering a specific tRNA, they share little similarity in nucleic acid sequence. Specifically, both their toxin and antitoxin components exhibit several markedly different features (summarized in Figure 7A and below).

Unlike the *H. hispanica creA* gene that contains only one CRISPR repeat-like (ΨR) sequence, the *H. hubeiense* antitoxin gene contains two ΨR sequences (ΨR1 and ΨR2) flanking the ΨS sequence. Accordingly, the mature *H. hispanica* CreA contains only the conserved 5′ handle, whereas the mature *H. hubeiense* CreA contains both the 5′ handle and the 3′ handle. By sequence similarity search, we found homologs of *H. hispanica* and *H. hubeiense creTA* in 4 and 12 haloarchaeal strains, respectively (Figure 7A). Importantly, by analyzing more archaeal and bacterial *creTA* modules predicted in our previous study, we showed that *creA* genes with two ΨR sequences are actually more common in CRISPR-Cas loci (Figure 3 and Supplementary Figure S2). However, in all these cases, ΨR1 and ΨR2 share very limited sequence identity, which should hinder their prediction and definition as a minimal CRISPR array. Furthermore, the two-ΨR *creA* genes lack the leader sequence that is required for CRISPR growth (6,31). These features explicitly distinguish *creA* from a typical CRISPR (mini)array. We also noted that, in most of the two-ΨR *creA* genes, the last 8 nucleotides of ΨR1 comprising the 5′ handle are more similar to the co-occurring CRISPR repeat than the respective nucleotides of ΨR2. Conversely, the first 22 nucleotides of ΨR2 comprising the 3′ handle are usually more similar to the CRISPR repeat than the corresponding portion of ΨR1 (Figures 1C and 3; Supplementary Figure S2). We surmise that ΨR1 and ΨR2 both evolved via duplications of the cognate CRISPR repeat, in an evolutionary scenario resembling that proposed for the evolution of the tracrRNA in type II CRISPR-Cas systems (32). Subsequently, ΨR1 and ΨR2 degenerated divergently by accumulating different point mutations within their respective less important regions, and when a transcriptional terminator was evolved to produce the 3′ terminus of CreA RNAs, ΨR2 may become more degenerated (like the case of *H. marismortui*; see Figure 3A) or completely lost (like the case of *H. hispanica* (17)) during evolution (Figure 7B). The divergent degeneration of two ΨR sequences prevented the loss of *creA* via recombination events, which frequently occur between canonical CRISPR repeats and contribute to CRISPR dynamics (33–35).

It would be interesting to explore why two-ΨR *creA* genes are more popular. We suppose that the two-handle CreA RNA molecules could be more stable, because the 3′ handle, which is tightly bound by Cas6 after cleavage (36) and serves as a nucleation point for Cascade assembly (37), proved to be critical for crRNA *in vivo* stability (24). To test this possibility, we added a second ΨR to the *H. hispanica creA*, and, conversely, replaced the second ΨR of *H. hubeiense creA* with a transcription terminator (Supplementary Figure S7A). By Northern blotting, we confirmed that, in both cases, the mature RNA products from two-ΨR *creA* was in much higher abundance than those from one-ΨR *creA* (Supplementary Figure S7B). Consistently, the P*_creT_*-repressing effect of two-ΨR *creA* was much stronger (Supplementary Figure S7C). Therefore, two-handle CreA antitoxins are favoured likely due to their higher stability and efficiency, especially when the toxin gene is driven by a strong promoter (*H. hubeiense* P*_creT_* appears to be ∼4.5-fold as strong as *H. hispanica* P*_creT_* according to the data from Supplementary Figure S7C).

*H. hispanica* and *H. hubeiense creT* genes each contains a mini-ORF, which consists of two consecutive minor codons of arginine or isoleucine, and a stem-loop structure located downstream of the mini-ORF (Figure 7A). Both mini-ORFs begin with the most efficient start codon AUG. *H. hispanica creT* also carries a strong SD motif that could enhance the efficiency of translation initiation. Our data in this study (Supplementary Figure S6) showed that the toxicity of CreT requires strong signals for translation initiation, which likely determines the efficiency and effect of tRNA sequestration. Interestingly, both mini-ORFs terminate with a conserved opal stop codon (UGA). Nevertheless, our mutational analysis showed that this opal stop codon is not essential for the activity of *H. hubeiense* CreT (Supplementary Figure S8). When UGA was mutated to another stop codon (UAA or UAG) or a sense codon like AGA, CGA, UCA or UGU, the pTA-Hhub derivates transformed *H. hispanica cas6-* cells with efficiencies that were 10^3^∼10^4^-fold reduced compared to the empty vector. As shown in Supplementary Figure S8, some mutations (e.g. to GGA, UUA, UGG or UGC) resulted in much smaller colonies on the screening plates although the transformation efficiency of the mutated plasmids reached the level of the empty vector. Therefore, although highly conserved, this opal stop codon is not essential for CreT function, suggesting the possibility that some still unidentified *creT* genes sequester a rare tRNA with minor codons located in the beginning of a longer ORF. However, we did not find such an ORF for another four haloarchaeal *creTA* genes (data not shown), suggesting distinct toxicity mechanisms.

In this and the previous studies (17), we characterized two groups of *creTA* modules that convergently sequester a specific rare tRNA, allowing us to infer some general and specific features of their toxins and antitoxins. The finding of the present work that *creA* more commonly carries two CRISPR repeat-like sequences is important for understanding the origin of CreTA from CRISPR repeats and the secondary, regulatory roles of Cas proteins. With the current, limited knowledge on the tRNA-sequestering small RNAs, it is difficult to predict the toxicity mechanism for other *creTA* modules, which highlights the diversity of CRISPR-regulated toxins. Structural and functional dissection of additional *creTA* modules can be expected to further enrich our knowledge of CRISPR biology and, particularly, the addictive properties of CRISPR-Cas mediated by associated toxin-antitoxin modules.

## DATA AVAILABILITY

The RNA sequencing data were deposited to the National Microbiology Data Center (NMDC) (https://nmdc.cn/resource/genomics/project) under the accession number NMDC10017848.

## Supplementary Material

gkab821_Supplemental_File
