# Supplementary material for: Divergent degeneration of creA antitoxin genes from minimal CRISPRs and the convergent strategy of tRNA-sequestering CreT toxins
Source: Nucleic Acids Res. 2021 Sep 22;49(18):10677–88. doi: 10.1093/nar/gkab821 (PMC8501985; doi:10.1093/nar/gkab821)
Supplement: gkab821_Supplemental_File [file gkab821_supplemental_file.pdf]

Supplementary material for：

Divergent degeneration of *creA* antitoxin genes from minimal CRISPRs and the convergent strategy of tRNA-sequestering CreT toxins

Feiyue Cheng[2,3], Rui Wang[4,1], Haiying Yu[2], Chao Liu[1], Jun Yang[1,5], Hua Xiang[2,3,*] and Ming Li[1,2,3,*]

[1]CAS Key Laboratory of Microbial Physiological and Metabolic Engineering, Institute of Microbiology, Chinese Academy of Sciences, Beijing, China.
[2]State Key Laboratory of Microbial Resources, Institute of Microbiology, Chinese Academy of Sciences, Beijing, China.
[3]College of Life Science, University of Chinese Academy of Sciences, Beijing, China.
[4]Non-coding RNA and Drug Discovery Key Laboratory of Sichuan Province, Chengdu Medical College, Chengdu, Sichuan, China
[5]Center for Life Science, School of Life Sciences, Yunnan University, Kunming, China

* To whom correspondence should be addressed. Tel: 010-64807064; Fax: 010-64807064; Email: lim_im@im.ac.cn; Correspondence can also be addressed to Hua Xiang. Email: xiangh@im.ac.cn

The file includes:

Figures S1 to S8
Tables S1 to S2
References

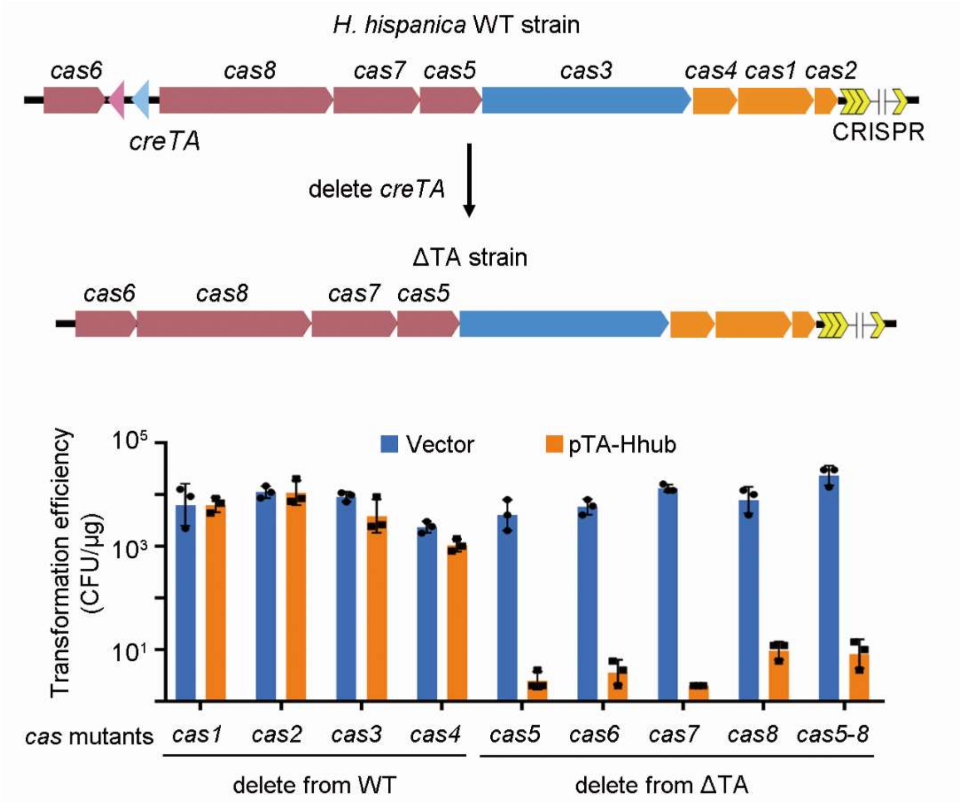

Figure S1 Toxicity of pTA-Hhub (carrying the *creTA* from *H. hubeiense*) in *H. hispanica* cells with different *cas* background. Note that, because the native *creTA* in *H. hispanica* cells can elicit dormancy or cell death when any of *cas5-8* genes are destroyed, single deletion mutants of these genes were constructed from a *H. hispanica* strain lacking its native *creTA* (ΔTA). When we were deleting *cas5-8* genes as a whole, the internal native *creTA* was simultaneously deleted. Error bars, mean ± s.d. (n = 3). Scattered dots indicate individual values.

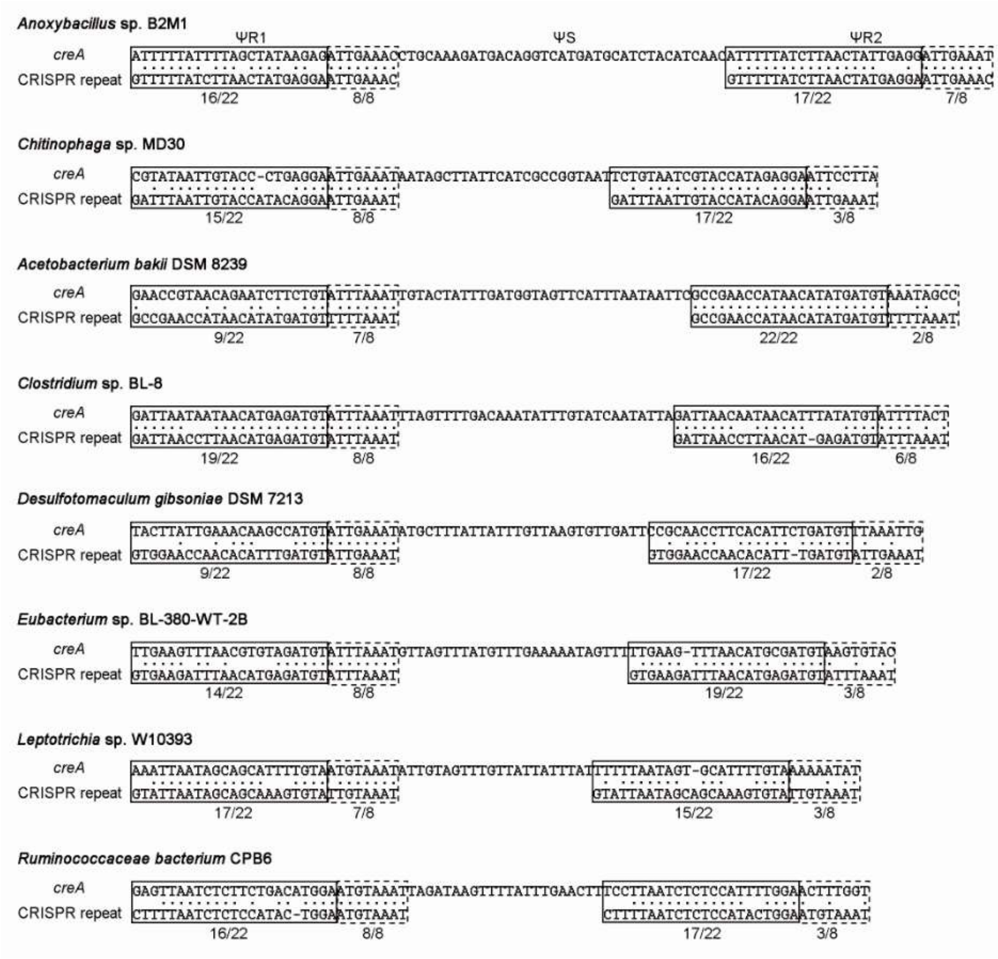

Figure S2 Bacterial *creA* analogs each has two CRISPR repeat-like units (ΨR1 and ΨR2) flanking a single spacer-like sequence (ΨS). Sequence identity between ΨR and the CRISPR repeat co-occurring in the same genome was calculated separately for the first 22 nucleotides and the last 8 nucleotides.

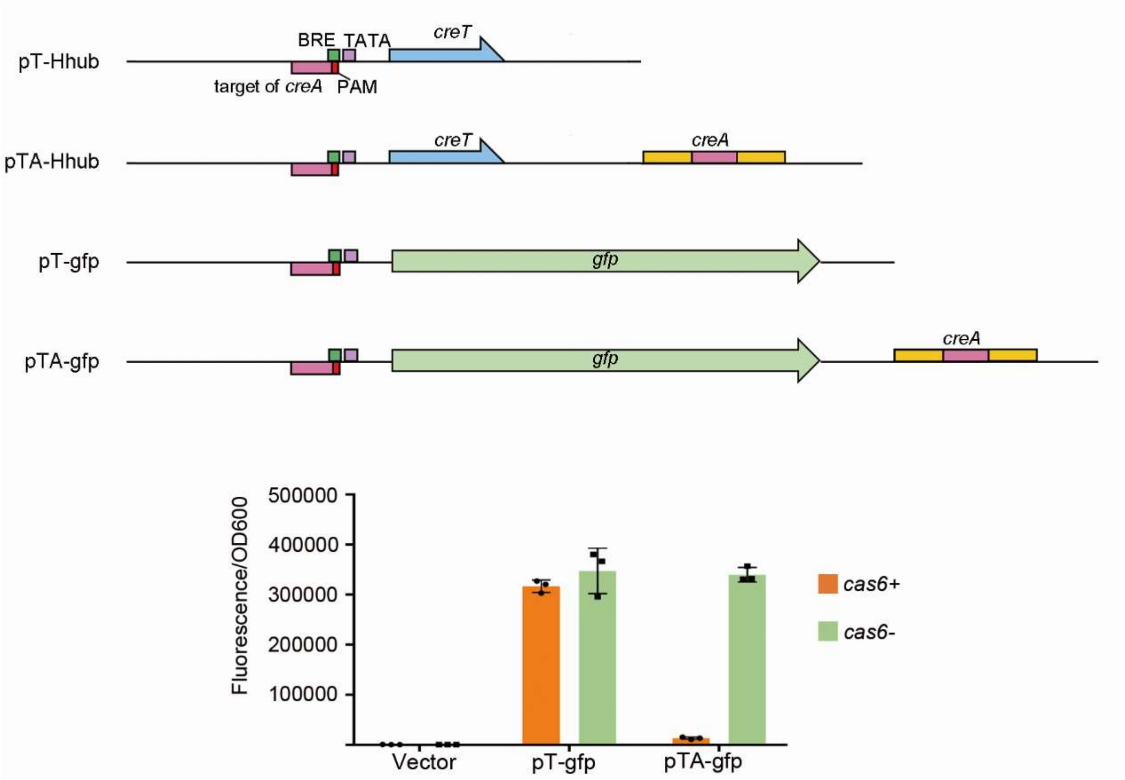

Figure S3 Fluorescence from a *gfp* reporter gene that is controlled by the *H. hubeiense* P_{creTA} with (pTA-gfp) or without (pT-gfp) the coexistence of its cognate *creA*. The protospacer target and PAM (protospacer adjacent motif) of *creA* overlap with the complement of the promoter element BRE. Fluorescence was monitored in *H. hispanica* cells encoding or lacking the Cas6 protein, and the optical density was synchronously determined. Error bars, mean ± s.d. (n = 3). Scattered dots indicate individual values.

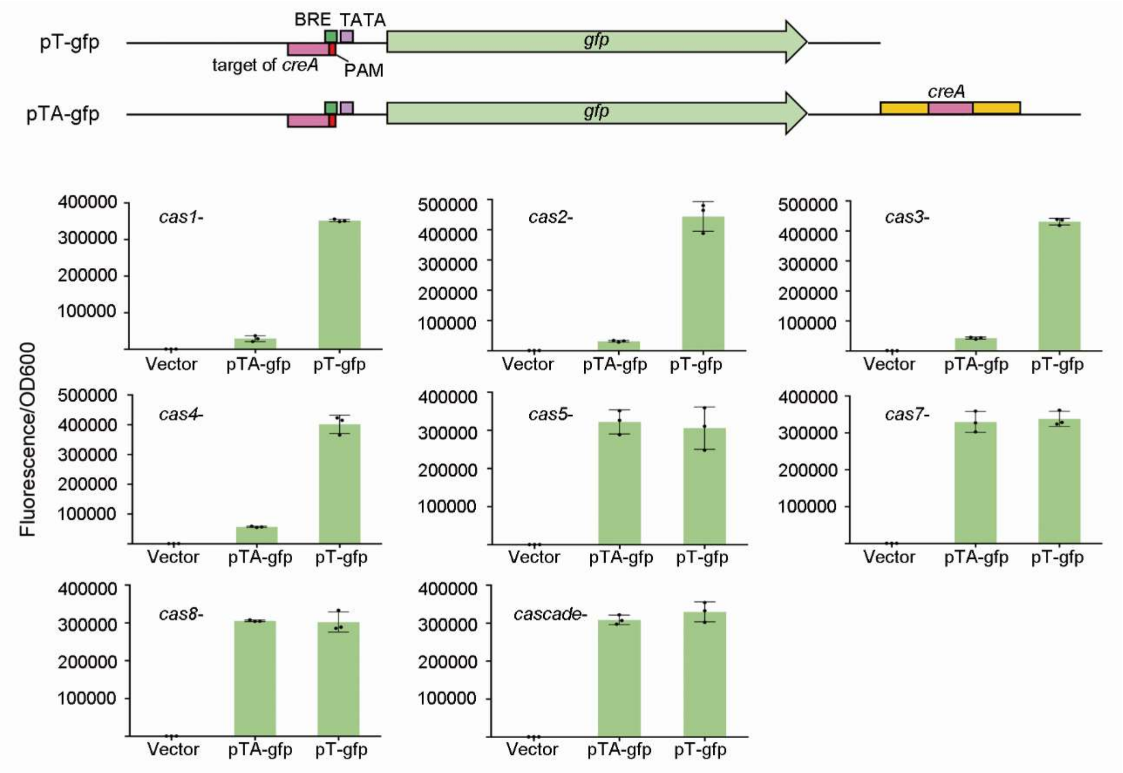

Figure S4 Fluorescence from a P_{creTA}-controlled *gfp* with (pTA-gfp) or without (pT-gfp) the coexistence of *creA* in *H. hispanica* cells lacking *cas1*, *cas2*, *cas3*, *cas4, cas5, cas7, cas8,* or all *cascade* genes. The protospacer target and PAM (protospacer adjacent motif) of *creA* overlap with the complement of the promoter element BRE. Fluorescence and the optical density were synchronously determined. Vector, the empty pWL502. Error bars, mean ± s.d. (n = 3). Scattered dots indicate individual values.

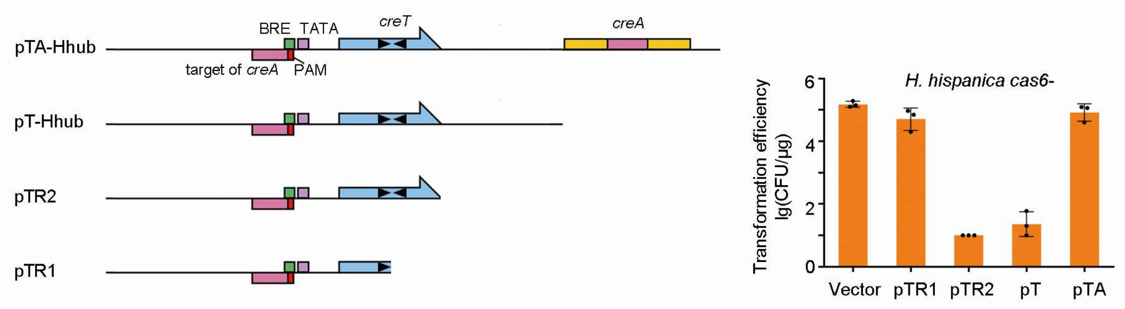

Figure S5 Truncation assay to analyze the importance of the inverted repeats (indicated with a pair of arrowheads) for *H. hubeiense creT* function. The protospacer target and PAM (protospacer adjacent motif) of *creA* overlap with the complement of the promoter element BRE. *H. hispanica cas6*- cells were transformed in triplicate. Error bars, mean ± s.d. (n = 3). Scattered dots indicate individual values.

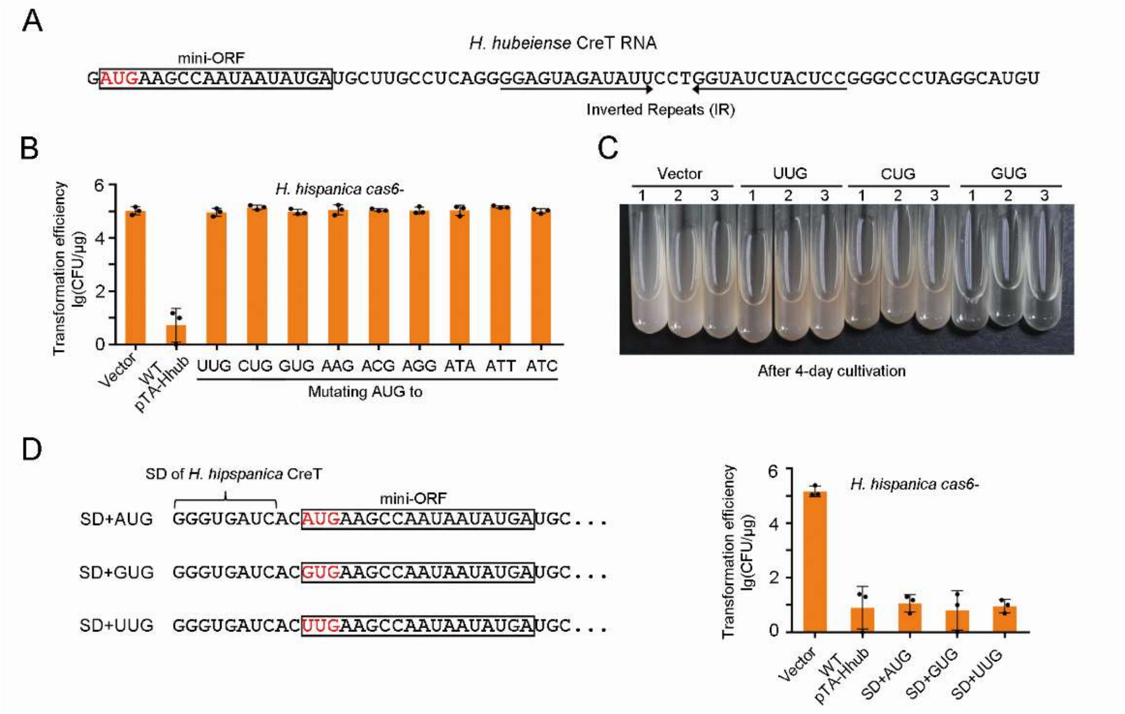

Figure S6 Saturation mutagenesis of the start codon of *H. hubeiense* CreT. (**A**) The sequence of *H. hubeiense* CreT RNA (with the start codon highlighted in red). (**B**) Efficiency of transforming the *H. hispanica cas6*-cells with WT or mutated pTA-Hhub. (**C**) Growth of some transformants in liquid medium. Three individual colonies were randomly selected for each plasmid. (**D**) Transforming *H. hispanica cas6*-cells with pTA-Hhub derivates that carry the Shine-Dalgarno (SD) sequence from *H. hispanica* CreT and one of the three start codons (AUG, GUG, and UUG). Error bars, mean ± s.d. (n = 3). Scattered dots indicate individual values.

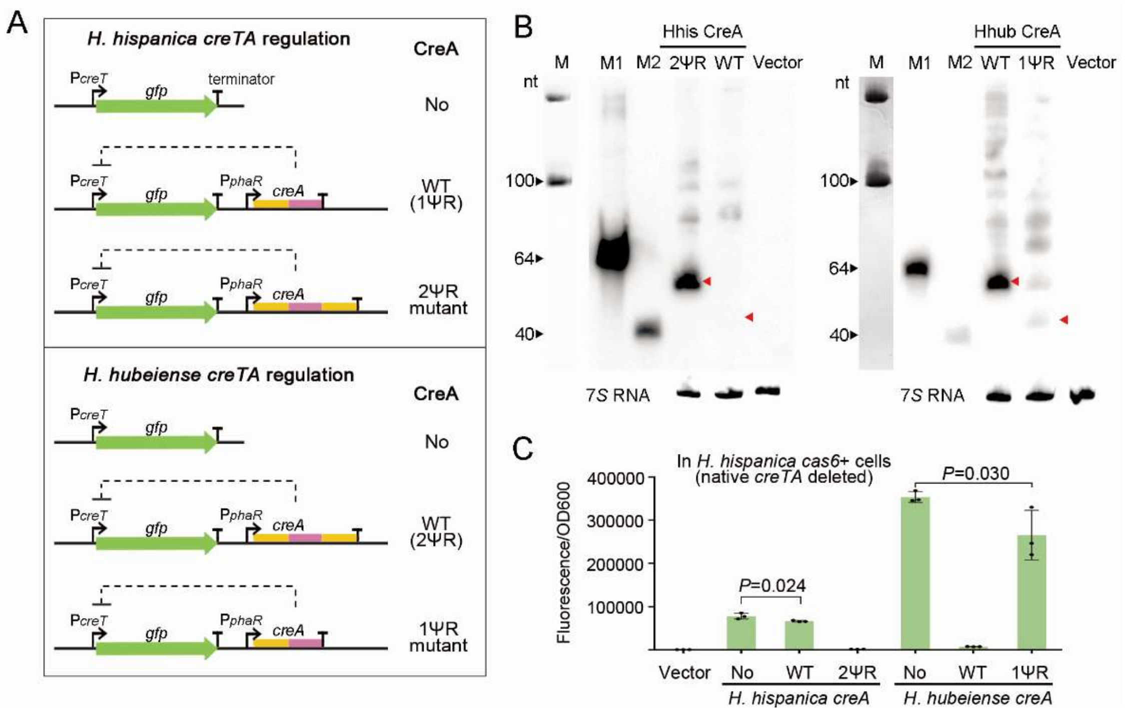

Figure S7 *In vivo* stability and P_{creT}-repressing effect of one- or two-handle CreA antitoxins. (**A**) Each plasmid carries a *gfp* gene that was controlled by *H. hispanica* or *H. hubeiense* P_{creT}. A second ΨR sequence was added to the WT *H. hispanica creA* to generate its two-ΨR mutant, and, conversely, the second ΨR of the WT *H. hubeiense creA* was replaced by a transcription terminator (eight consecutive thymines) to generate its one-ΨR mutant. P_{phaR}, a strong constitutive promoter. (**B**) Northern blotting of CreA molecules from plasmids in panel **A** in the *H. hispanica cas6*+ cells where the native *creTA* module was deleted. Red triangles indicate the predicted positions of the RNA band of mature CreA. M, 100-nt RNA ladder. M1 and M2, biotin-labeled 40-nt and 64-nt ssDNA, respectively. (**C**) Fluorescence from plasmids in panel A. Error bars, mean ± s.d. (n = 3). Scattered dots indicate individual values. *P* values, one-tailed Student's t test.

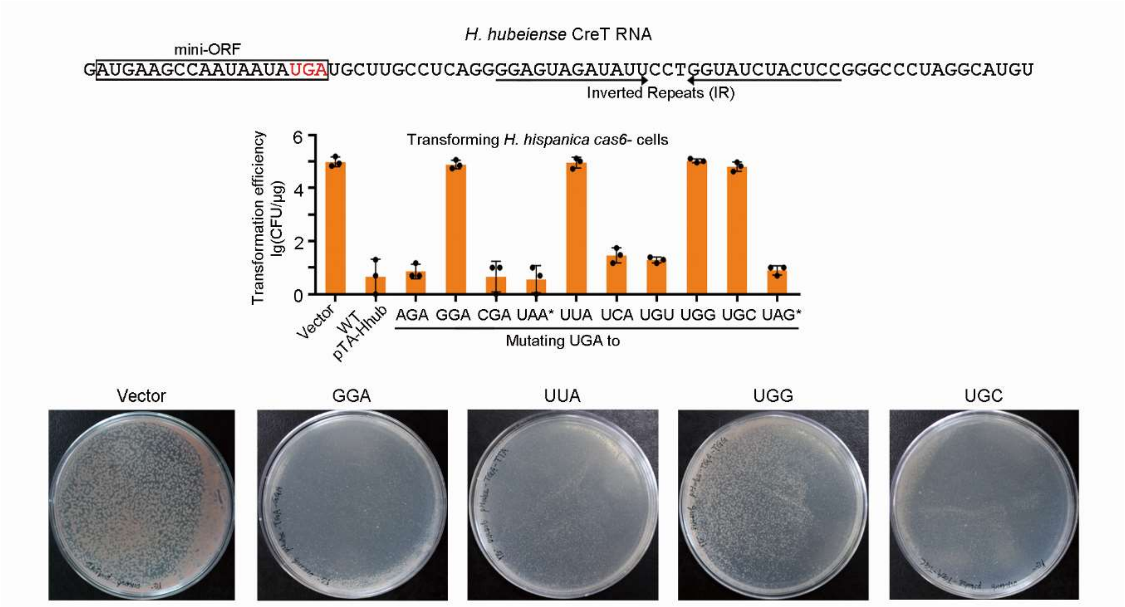

Figure S8 Mutagenesis of the stop codon (UGA in red) of *H. hubeiense* CreT. The histogram summarizes the efficiency when transforming the *cas6*-deleted *H. hispanica* cells with the WT or mutated pTA-Hhub. Images in the bottom show the much smaller colonies of pTA-Hhub transformants when UGA was mutated to GGA, UUA, UGG, or UGC. Stop codons are labeled with *. Error bars, mean ± s.d. (n = 3). Scattered dots indicate individual values.

Table S1 Strains used in this study.

Strain Name	Description	References
DF60	*pyrF* deletion mutant of *H. hispanica* ATCC 33960	(1)
cas6+ (ΔTA)	*creTA* (native) deletion mutant of *H. hispanica* DF60	(2)
cas5-	*cas5* deletion mutant of ΔTA	
cas6-	*cas6* deletion mutant of ΔTA	
cas7-	*cas7* deletion mutant of ΔTA	
cas8-	*cas8* deletion mutant of ΔTA	
Δ*cas5-8*	Cascade-encoding genes (*cas5-8*) deletion mutant of DF60 (the native *creTA* between *cas6* and *cas8* was also deleted)	
Δ*cas1*	*cas1* deletion mutant of DF60	
Δ*cas2*	*cas2* deletion mutant of DF60	
Δ*cas3*	*cas3* deletion mutant of DF60	
Δ*cas4*	*cas4* deletion mutant of DF60	
H37A	*cas6* dead mutant of ΔTA	

Table S2 Oligonucleotides used in this study.

Name	Sequence (5′—3′) [a]	Description
**For pTA-Hhub construction & truncation**		
TA-Hhub-F	CGCGGATCCGGGTACGTTTCAGACGCACC	Forward primer for TA amplification
TA-Hhub-R	CGGGGTACCAGTGTTATGTCGATTTAGGT	Reverse primer for TA amplification
T-Hhub-R	CGGGGTACCAGATTGGGTGCCTCGTCAAG	Reverse primer for TA truncation
TA-R	CGGGGTACCTGATGAGAAGTACATGCC	Reverse primer for TA truncation
TR1-R	CGGGGTACCGGAATATCTACTCCCCTGAG	Reverse primer for TA truncation
TR2-R	CGGGGTACCACATGCCTAGGGCCCGGA	Reverse primer for TA truncation
**For P_{phaR}-*creA* construction & *creA* mutation**		
phaR-creA-F1	CGCGGATCCAATGGTGTCGAAGGGAACATATATGTTACTGCAGGTACAACAC	Forward primer for *creA* amplification; containing partial sequence of P_{phaR} for extension
phaR-CreA-F2	TGTTACTGCAGGTACAACACCGAGTTAGGAGATGGTATCAGATGAACGCAAC	Forward primer for *creA* amplification; containing partial sequence of P_{phaR} for extension
Hhub-creAm01-F	AACTGCGGTTGAAGT***G***ACCAGTATGGTGCCA	T1G
Hhub-creAm01-R	TGGCACCATACTGGT***C***ACTTCAACCGCAGTT	T1G
Hhub-creAm02-F	AACTGCGGTTGAAGTT***C***CCAGTATGGTGCCAAC	A2C
Hhub-creAm02-R	GTTGGCACCATACTGG***G***AACTTCAACCGCAGTT	A2C
Hhub-creAm03-F	CTGCGGTTGAAGTTA***A***CAGTATGGTGCCAAC	C3A
Hhub-creAm03-R	GTTGGCACCATACTG***T***TAACTTCAACCGCAG	C3A
Hhub-creAm04-F	TGCGGTTGAAGTTAC***A***AGTATGGTGCCAACACCGT	C4A
Hhub-creAm04-R	ACGGTGTTGGCACCATACT***T***GTAACTTCAACCGCA	C4A
Hhub-creAm05-F	GCGGTTGAAGTTACC***C***GTATGGTGCCAACAC	A5C
Hhub-creAm05-R	GTGTTGGCACCATAC***G***GGTAACTTCAACCGC	A5C
Hhub-creAm06-F	GCGGTTGAAGTTACCA***T***TATGGTGCCAACAC	G6T
Hhub-creAm06-R	GTGTTGGCACCATA***A***TGGTAACTTCAACCGC	G6T
Hhub-creAm07-F	GGTTGAAGTTACCAG***G***ATGGTGCCAACACCG	T7G
Hhub-creAm07-R	CGGTGTTGGCACCAT***C***CTGGTAACTTCAACC	T7G
Hhub-creAm08-F	GTTGAAGTTACCAGT***C***TGGTGCCAACACCGT	A8C

Hhub-creAm08-R	ACGGTGTTGGCACCA***G***ACTGGTAACTTCAAC	A8C
Hhub-creAm09-F	TTGAAGTTACCAGTA***G***GGTGCCAACACCGTC	T9G
Hhub-creAm09-R	GACGGTGTTGGCACC***C***TACTGGTAACTTCAA	T9G
Hhub-creAm10-F	TTGAAGTTACCAGTAT***T***GTGCCAACACCGTCAC	G10T
Hhub-creAm10-R	GTGACGGTGTTGGCAC***A***ATACTGGTAACTTCAA	G10T
Hhub-creAm11-F	GAAGTTACCAGTATG***T***TGCCAACACCGTCAC	G11T
Hhub-creAm11-R	GTGACGGTGTTGGCA***A***CATACTGGTAACTTC	G11T
Hhub-creAm12-F	GAAGTTACCAGTATGG***G***GCCAACACCGTCAC	T12G
Hhub-creAm12-R	GTGACGGTGTTGGC***C***CCATACTGGTAACTTC	T12G
**For Northern blotting**		
Hhub-ψS probe	GACGGTGTTGGCACCATACTG	biotin-labeled; for Northern analysis
Hhis-ψS-probe	GATCGGGACAATAGCCCAAG	biotin-labeled; for Northern analysis
Hhis-7S probe	CGTCAGACCGGACGCCCCC	biotin-labeled; for Northern analysis
**For P_{creT} mutation**		
Hhub-P_{creT}-m4-F	GCAGTTATGCCATATT***T***GTAGAAAGAACTAAGT	C4A
Hhub-P_{creT}-m4-R	ACTTAGTTCTTTCTAC***A***AATATGGCATAACTGC	C4A
Hhub-P_{creT}-m10-F	GGGTGTGCAGTTATGC***A***ATATTGGTAGAAAGAA	G10T
Hhub-P_{creT}-m10-R	TTCTTTCTACCAATAT***T***GCATAACTGCACACCC	G10T
**For pTA-Hhub mutation**		
TA-Hhub-IRm-F	AGTAGATATTCCTGGT***CG***CT***CA***TCCGGGCCCTAGGCAT	Mutating one of the IRs
TA-Hhub-IRm-R	ATGCCTAGGGCCCGGA***TG***AG***CG***ACCAGGAATATCTACT	Mutating one of the IRs
TA-Hhub-IRcm-F	TGCTTGCCTCAGGGGA***TG***AG***CG***ATTCCTGGT***CG***CT***CA***TCCGGGCCCTAGGCAT	Complementarily mutating the IRs
TA-Hhub-IRcm-R	ATGCCTAGGGCCCGGA***TG***AG***CG***ACCAGGAAT***CG***CT***CA***TCCCCTGAGGCAAGCA	Complementarily mutating the IRs
**For CreT mutation**		
Hhub-AUG1m-F	ACAGCGGGGAAATAGGCTGTTG***B***TGAAGCCAATAATATG	mutating AUG to UUG/GUG/CUG
Hhub-AUG2m-F	ACAGCGGGGAAATAGGCTGTTGA***V***GAAGCCAATAATATGA	mutating AUG to AAG/ACG/AGG
Hhub-AUG3m-F	ACAGCGGGGAAATAGGCTGTTGAT***H***AAGCCAATAATATGATG	mutating AUG to AUA/AUC/AUU
Hhub-AUGm-R	CAACAGCCTATTTCCCCGCTGT	mutating AUG
Hhub-UGA1m-F	GCTGTTGATGAAGCCAATAATA***V***GATGCTTGCCTCAGGG	mutating UGA to AGA/CGA/GGA

Hhub-UGA2m-F	GCTGTTGATGAAGCCAATAATAT***H***ATGCTTGCCTCAGGGG	mutating UGA to UAA/UUA/UCA
Hhub-UGA3m-F	GCTGTTGATGAAGCCAATAATATG***B***TGCTTGCCTCAGGGGA	mutating UGA to UGU/UGC/UGG
Hhub-UGAm-R	TATTATTGGCTTCATCAACAGC	mutating UGA
Hhub-UAG-F	GATGAAGCCAATAATA***TAG***TGCTTGCCTCAGGGGA	mutating UGA to UAG
Hhub-UAG-R	TCCCCTGAGGCAAGCA***CTA***TATTATTGGCTTCATC	mutating UGA to UAG
AUA-AUC-F	CTGTTGATGAAGCCA***ATCATC***TGATGCTTGCCTCAGG	mutating the two AUA codons to AUC
AUA-AUC-R	CCTGAGGCAAGCATCA***GATGAT***TGGCTTCATCAACAG	mutating the two AUA codons to AUC
AUA-AUU-F	CTGTTGATGAAGCCA***ATTATT***TGATGCTTGCCTCAGG	mutating the two AUA codons to AUU
AUA-AUU-R	CCTGAGGCAAGCATCA***AATAAT***TGGCTTCATCAACAG	mutating the two AUA codons to AUU
AUA-AGA-F	GAAATAGGCTGTTGATGAAGCCA***AGAAGA***TGATGCTTGCCTCAG	mutating the two AUA codons to AGA
AUA-AGA-R	CTGAGGCAAGCATCA***TCTTCT***TGGCTTCATCAACAGCCTATTTC	mutating the two AUA codons to AGA
AUA-AGG-F	GAAATAGGCTGTTGATGAAGCCA***AGGAGG***TGATGCTTGCCTCAGG	mutating the two AUA codons to AGG
AUA-AGG-R	CCTGAGGCAAGCATCA***CCTCCT***TGGCTTCATCAACAGCCTATTTC	mutating the two AUA codons to AGG
AAGCCA-F	TAGGCTGTTGATGATAATATGATGCTTGCCTCAGGGGA	deleting AAG and CCA codons
AAGCCA-R	TCATATTATCATCAACAGCCTATTTCCCCGCT	deleting AAG and CCA codons
SDAUG-F	CTGTTG***GGGTGATC***ACATGAAGCCAATAATATGATGCTTGC	adding SD motif
SDAUG-R	CTTCATGT***GATCACCC***CAACAGCCTATTTCCCCGCTGTCA	adding SD motif
SDGUG-F	CTGTTG***GGGTGATC***AC***GTG***AAGCCAATAATATGATGCTTGC	mutating AUG to GUG; adding SD motif
SDGUG-R	CTT***CAC***GT***GATCACCC***CAACAGCCTATTTCCCCGCTGTCA	mutating AUG to GUG; adding SD motif
SDUUG-F	CTGTTG***GGGTGATC***AC***TTG***AAGCCAATAATATGATGCTTGC	mutating AUG to UUG; adding SD motif
SDUUG-R	CTT***CAA***GT***GATCACCC***CAACAGCCTATTTCCCCGCTGTCA	mutating AUG to UUG; adding SD motif
**For tRNA overexpression**		
TA-tRNA-UR	<u>AGTAACATATATGTTCCCTTCGAGTGTTATGTCGATTTAGGTAT</u>	linking *creTA* to a tRNA gene; containing partial sequence of P_{phaR}
TA-tRNA-F	<u>ATACCTAAATCGACATAACACT</u>CGAAGGGAACATATATGTTACT	linking *creTA* to a tRNA gene; containing partial sequence of P_{phaR}
TA-tRNA^{UCU}-R	CGG<u>GGTACC</u>TCTGGCGTGATTCGAATCCC	amplifying the tRNA^{UCU} gene
TA-tRNA^{CCU}-R	CGG<u>GGTACC</u>CTCGTCTCGTAGGGTTCGAATCT	amplifying the tRNA^{CCU} gene

TA-tRNA^{CAU}-F	CGAAGGGAACATATATGTTACTGCAGGTACAACACCGAGTTAAACCCCTCCACTCCAACCCGACA	linking *creTA* to the tRNA^{CAU} gene; containing partial sequence of P_{phaR}
TA-tRNA^{CAU}-R	CGGGGTACCAAAAAAAAGAGTTGTGGCGTGTGAGTC	amplifying the tRNA^{CAU} gene
**For GFP expression**		
TA-gfp-R	GAAAAGTTCTTCTCCTTTACTCATCAACAGCCTATTTCCCCGCTG	linking *creTA* to the *gfp* gene
gfp-F	ATGAGTAAAGGAGAAGAACTTTT	amplifying the *gfp* gene
gfp-R	GCATTATTTGTATAGTTCATCCATGCC	amplifying the *gfp* gene
TA-gfp-F	GCATGGATGAACTATACAAATAATGCTTGCCTCAGGGGAGTAGA	linking *creTA* to the *gfp* gene
gfp-R2	CGGGGTACCAAAAAAAATTATTTGTATAGTTCATCCATGC	amplifying the *gfp* gene
Hhis-P_{creT}-F	CGCGGATCCTGGTTGGCCACATAGTGACGTC	Forward primer for *Haloarcula hispanica* P_{creT} amplification
Hhis-P_{creT}-R	TGAAAAGTTCTTCTCCTTTACTCATGTGATCACCCTTCTTGTCAA	Reverse primer for *Haloarcula hispanica* P_{creT} amplification; containing partial sequence of *gfp* for extension
gfp-phaR-R	CAGTAACATATATGTTCCCTTCGAAAAAAAATTATTTGTATAGTTCATCCATGC	amplifying the *gfp* gene; containing partial sequence of P_{phaR} for extension
Hhis-gfp-creA-F	CGAAGGGAACATATATGTTACTGCAGGTACAACACCGAGTTAGGAGATTCCAGATGAACAAAGGTTGGGTTGAAGT	amplifying the *Haloarcula hispanica creA* gene
Hhis-creA1-R	CGGGGTACCAAAATGAACTGGGATCGGGACAATAGCCCAAGGACTTCAACCCAACCTTTGTTCAT	amplifying the *Haloarcula hispanica creA* gene
Hhis-creA2-R	CGGGGTACCAAAAAAAAGCTTCAACCCCACGAGGGTTCGTCTGAAACCTGGGATCGGGACAATAGCCCA	amplifying the *Haloarcula hispanica* modified *creA* gene
Hhub-creA-F	CGAAGGGAACATATATGTTACTGCAGGTACAACACCGAGTTAGGAGGTATCAGATGAACGCAACTGCGGTTGAAGT	amplifying the *creA* gene
Hhub-creA1-R	CGGGGTACCAAAAAAAATGATGAGAAGTACATGCCAGCACCC	amplifying the *creA* gene
Hhub-creA2-R	CGGGGTACCAAAAAAAATTGTGACGGTGTTGGCACCATACTGGTAACTTCAACCGCAGTTGCGTTCA	amplifying the modified *creA* gene

[a]underlined, restriction sites; F, forward primer; R, reverse primer; double underlined, overlapping sequences designed for overlap extension PCR; bold and italicized, mutated nucleotides.

REFERENCES

1. Liu,H., Han,J., Liu,X., Zhou,J. and Xiang,H. (2011) Development of *pyrF*-based gene knockout systems for genome-wide manipulation of the archaea *Haloferax mediterranei* and *Haloarcula hispanica*. *J. Genet. Genomics*, **38**, 261-269.
2. Li,M., Gong,L., Cheng,F., Yu,H., Zhao,D., Wang,R., Wang,T., Zhang,S., Zhou,J., Shmakov,S.A. *et al.* (2021) Toxin-antitoxin RNA pairs safeguard CRISPR-Cas systems. *Science*, **372**, eabe5601.
